# In silico investigation of BC_59_ nanocage for furan adsorption and single-use detection potential

**DOI:** 10.3389/fchem.2026.1913300

**Published:** 2026-09-30

**Authors:** Bader Huwaimel, Saad Alqarni, Ali Alghubayshi, Talal Alotaibi, Hashem O. Alsaab, Rami M. Alzhrani, Amr S. Abouzied

**Affiliations:** 1 Department of Pharmaceutical Chemistry, College of Pharmacy, University of Ha’il, Ha’il, Saudi Arabia; 2 Medical and Diagnostic Research Center, University of Ha’il, Hail, Saudi Arabia; 3 Department of Clinical Pharmacy, College of Pharmacy, University of Hail, Hail, Saudi Arabia; 4 Department of Pharmaceutics and Industrial Pharmacy, College of Pharmacy, Taif University, Taif, Saudi Arabia

**Keywords:** boron doping, density functional theory, fullerene C_60_, furan adsorption, permanent adsorbent, single-use detection platform

## Abstract

Furan is a hazardous environmental and food-processing contaminant for which rapid and accessible detection methods are needed. In this theoretical density functional theory study, pristine C_60_ and its B- and N-substituted derivatives, BC_59_ and NC_59_, were evaluated as candidate materials for furan adsorption and sensing-related applications using the B97D and ωB97X-D functionals. The two functionals produced consistent qualitative rankings for the calculated properties, with Pearson correlation coefficients greater than 0.97 and Spearman coefficients of 1.00. Among the investigated systems, BC_59_ exhibited the strongest calculated adsorption, with adsorption energies ranging from −34.61 to −49.44 kcal.mol^−1^, together with the largest adsorption-induced conductivity change and a pronounced reduction in the HOMO-LUMO energy gap. NBO analysis indicated stronger donor-acceptor stabilization in BC_59_@Furan, with E^(2)^ values of up to 61.81 kcal.mol^−1^. QTAIM, NCI, RDG, ELF, and LOL analyses also suggested a stronger and more localized interaction at the BC_59_@furan interface than in the C_60_@Furan and NC_59_@Furan complexes. However, the exceptionally long predicted desorption times indicate that BC_59_ would not be suitable for rapidly reusable sensing applications at room temperature. Instead, it may be considered a candidate permanent adsorbent or a theoretically proposed single-use detection platform. These computational predictions represent a pre-synthesis screening step and require experimental validation before practical application.

## 1 Introduction

Furan is a volatile organic compound formed during the thermal degradation of naturally occurring substances. It is found in several heat-processed foods and may also be released through tobacco smoke, combustion, and industrial activities (Bakhiya and Appel, 2010; Kirkok et al., 2020). Because furan is considered a potential carcinogenic contaminant, reliable monitoring methods are needed to reduce exposure, optimize food-processing conditions, and support compliance with food-safety regulations (Batool et al., 2023; Mariotti et al., 2013). In addition to its relevance as an environmental and food contaminant, furan has been investigated as a potential non-invasive biomarker for prostate cancer (Jiménez-Pacheco et al., 2018). This dual relevance increases the need for accurate analytical determination. Conventional methods, including gas chromatography-mass spectrometry, high-performance liquid chromatography, and solid-phase microextraction, can provide sensitive measurements. However, their cost, operational complexity, and requirement for specially trained personnel may limit their use in routine or portable monitoring (Altaki et al., 2007; Goldmann et al., 2005).

The determination of furan is challenging because of its volatility and thermal instability, which may contribute to analyte loss and reduced reproducibility during sample preparation and measurement. Its relatively low polarity may also require carefully optimized extraction and analytical procedures. These limitations motivate the investigation of complementary nanomaterial-based approaches that could potentially support direct, portable, and room-temperature detection (Uchida et al., 2018). Recent studies have investigated electrochemical and colorimetric platforms as alternatives or complements to conventional analytical methods (Hadi and Safari, 2022). Such platforms may offer advantages including portability, rapid response, and relatively low operating cost. Carbon-based nanomaterials, such as graphene, carbon nanotubes, and fullerenes, are of particular interest because their electronic structures and surface properties can be modified to promote analyte adsorption and adsorption-induced signal changes (Gaur et al., 2021; Power et al., 2018; Yusof et al., 2019). C_60_ fullerene has therefore been investigated as a component of several candidate sensing platforms because of its electron-accepting properties and accessible molecular surface (Yadav et al., 2026).

C_60_-based materials have been investigated for electrochemical, piezoelectric, and related sensing applications (Goyal et al., 2008; Shih et al., 2001). For example, Rather et al. developed a C_60_-based approach for bisphenol A detection and removal (Rather and De Wael, 2013). Other theoretical studies have examined metal-decorated C_60_ structures for interactions with pharmaceutical compounds and volatile organic molecules (Bassey et al., 2024; Ogunwale et al., 2022). Collectively, these studies support further investigation of fullerene-based materials as candidate platforms for environmental and biomedical analysis. Substitutional doping of C_60_ with heteroatoms such as boron or nitrogen can also modify its local geometry, electron distribution, Frontier-orbital energies, and affinity toward adsorbed molecules (Chen et al., 2001; Gao et al., 2013). Nattagh et al., for example, reported that B and N substitution altered the calculated adsorption behavior of C_60_ toward aspirin (Nattagh et al., 2021). These findings suggest that heteroatom substitution may provide a useful strategy for tuning the response of fullerene-based materials toward furan.

The present study theoretically compares pristine C_60_, BC_59_, and NC_59_ as candidate materials for furan adsorption and adsorption-induced electronic response. Calculations using density functional theory (DFT) and quantum theory of atoms in molecules (QTAIM), along with complementary analyses of electronic structure and electron density, were employed to identify qualitative trends and prioritize candidates for future synthesis and electrochemical evaluation (Cortés-Guzmán et al., 2023; Orio et al., 2009). The results are intended as a pre-synthesis screening step rather than as experimental validation of practical sensing performance.

## 2 Computational methods

In this study, the molecular structures of furan, pristine C_60_ fullerene, and their boron- and nitrogen-doped derivatives (BC_59_ and NC_59_), as well as their respective complexes, were initially constructed using GaussView 6.0 software (Figure 1A; Dennington et al., 2016). To obtain the most stable geometries, all designed structures underwent full geometric optimization using Gaussian 09W software (Frisch, 2009). Supplementary Figure S1 (In Supplementary Data) shows a rapid decrease in the total energy of C_60_, BC_59_, NC_59_, and furan during the initial optimization steps, followed by a nearly constant energy profile. This convergence indicates that all structures reached stable optimized geometries corresponding to local energy minima. To ensure reproducibility of the results, the XYZ coordinates of the designed structures were reported in Supplementary Table S1 in the Supplementary Data. All molecular geometries were fully optimized using the dispersion-modified B97D functional with the 6-31G(d) basis set. This level of theory was selected because B97D incorporates empirical dispersion corrections that are important for describing the weak van der Waals and π-related interactions expected in fullerene-based complexes (Grimme, 2006). Furthermore, Avramopoulos et al. previously applied the B97D/6-31G(d) level to optimize C_60_-containing complexes and showed that the 6-31G(d) basis set provided qualitatively satisfactory results. They also reported a B97D/6-31G(d) HOMO-LUMO gap of 1.66 eV for pristine C_60_, which was close to the experimental value of 1.85 ± 0.04 eV, supporting the applicability of this computational level to C_60_-based systems (Avramopoulos et al., 2016). The optimizations of all the calculated structures were performed in the water phase using the conductor-like polarizable continuum model (CPCM) of accounting for solvent effects (Samavat et al., 2021). Frequency calculations were performed at the same level of theory. The absence of imaginary vibrational frequencies confirmed that the optimized geometries correspond to local minima on the calculated potential-energy surface. This result does not, by itself, establish experimental stability or synthesizability.

**FIGURE 1:**
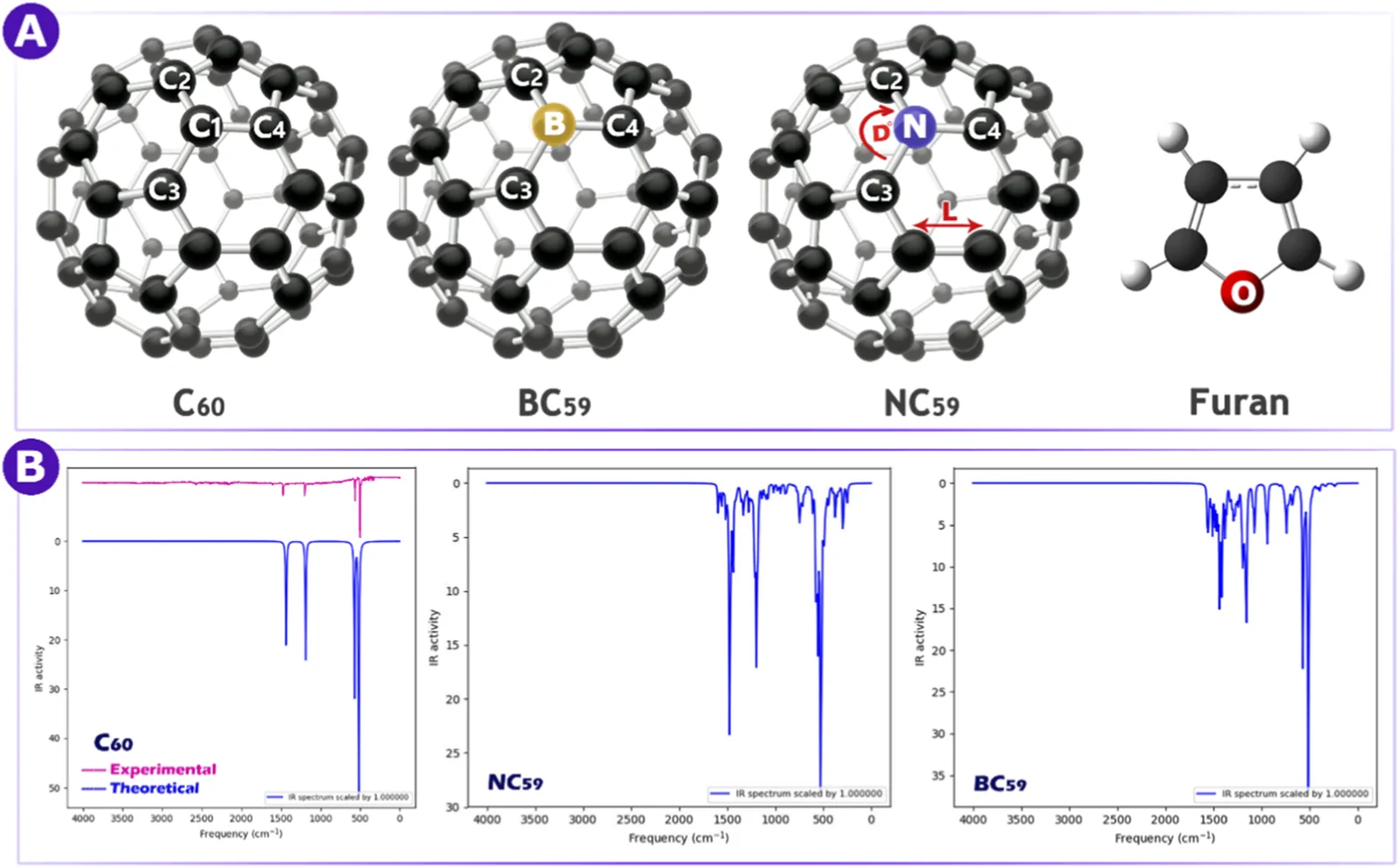
**(A)** Optimized geometry: furan, pristine C_60_, BC_59_, and NC_59_. **(B)** IR spectrum of C_60_ before/after doping with B (BC_59_) and N (NC_59_). Calculated using the B97D functional.

To examine the robustness of the predicted trends, the calculations were repeated using the ωB97X-D/6–31G(d) level of theory. The ωB97X-D functional includes long-range exchange and empirical dispersion corrections and was therefore used as an independent computational approach for comparing the relative adsorption and electronic-response trends (Chai and Head-Gordon, 2008). Both B97-D and ωB97X-D contain empirical atom-pair dispersion treatments, although these formulations are distinct from the later D3 correction and its associated damping schemes. A separate D3-based benchmark was not performed because a fully consistent comparison would require repeating the geometry optimizations, frequency calculations, adsorption-energy calculations, and subsequent electronic and topological analyses for all isolated structures and complexes. The absence of such a benchmark is therefore recognized as a methodological limitation. Agreement between B97-D and ωB97X-D is interpreted only as support for the robustness of the qualitative rankings and not as proof of quantitative accuracy or experimental validation. To quantify this consistency, Pearson correlation coefficients (r) and Spearman rank correlation coefficients (ρ) were calculated, as reported in Supplementary Table S2 and Supplementary Figure S2 of the Supplementary Data.

Stability was assessed by the cohesive energy (E_Coh_) (Equation 1).

$$E_{Coh}=E_{tot}-\sum_{i}n_{i}E_{i}/n$$ (1)

where E_Coh_ is the cohesive energy per atom, E_tot_ is the total electronic energy of the optimized structure, Ei is the electronic energy of an isolated atom of element i, n_i_ is the number of atoms of element i, and n is the total number of atoms in the structure. Under the adopted convention, a more negative E_Coh_ value indicates stronger average binding of the constituent atoms. The results are reported in kcal.mol^−1^ atom^−1^ and are used only for comparative purposes. It was not interpreted as definitive evidence of dopant synthesizability or thermodynamic accessibility (Hadi and Shamlouei, 2021).

The electronic properties and reactivity of each structure were evaluated by quantum descriptors (energy gap (E_gap_), chemical softness (S), chemical hardness (η), chemical potential (μ), maximum charge transfer value (ΔNmax), and electrophilicity-based charge transfer (ECT) (Equations 2–7)) (Cardenas-Jiron et al., 1997; Padmanabhan et al., 2007).

$$Egap=\left|E_{\text{HOMO}}-E_{\text{LUMO}}\right|$$ (2)

$$\eta =\left(-E_{\text{HOMO}}-\left(-E_{\text{LUMO}}\;\right)\right)/2$$ (3)

$$\mu =-\left(-E_{\text{HOMO}}+\left(-E_{\text{LUMO}}\right)\right)/2$$ (4)

S=1/2η (5)

ΔNmax=−μη (6)

$$ECT={\left({\Delta N}_{\max}\right)}_{\alpha }-{\left({\Delta N}_{\max}\right)}_{\beta }$$ (7)

In these equations, EHOMO and ELUMO are the Frontier-orbital energies, while Egap (in eV), η (in eV), S (in eV^−1^), μ (in eV), and ΔNmax (in e) denote the energy gap, chemical hardness, chemical softness, chemical potential, and maximum charge-transfer capacity, respectively. ECT was interpreted only as a qualitative indicator of the predicted charge-transfer direction. According to the adopted convention, a negative ECT value indicates electron transfer from furan toward the adsorbent, whereas a positive value indicates the reverse direction. The ECT magnitude was not interpreted as the amount or strength of transferred charge.

To compare the adsorption strength, predicted desorption behavior, and adsorption-induced electronic response, the adsorption energy (Eads), recovery time (τ), and conductivity-related quantity (σ) were calculated using Equations 8–10, respectively. These parameters were used to compare the interaction strength, predicted desorption behavior, and adsorption-induced electronic response of the investigated systems (Yadav et al., 2023).

$$E_{ads}=E_{Surface@Furan}-\left(E_{Furan}+E_{Surface}\right)+E_{BSSE}$$ (8)

$$\tau =V_{0}^{-1}\times \exp\left(-\frac{E_{ads}}{k_{B}T}\right)$$ (9)

$$\sigma =AT^{3/2}e^{\left(-E\text{gap}/2k_{B}T\right)}$$ (10)

where Eads is the adsorption energy in kcal.mol^−1^; E_Surface@Furan_ is the total electronic energy of the optimized surface-furan complex; E_Surface_ is the total electronic energy of the isolated surface, namely C_60_, BC_59_, or NC_59_; and E_Furan_ is the total electronic energy of the isolated furan molecule. All three electronic energies in Equation 8 were calculated at the same level of theory and expressed in the same energy unit. A negative Eads value denotes an energetically favorable adsorption process according to the adopted convention. Also, E_BSSE_ is the basis-set-superposition-error correction obtained using the counterpoise procedure. It is included to reduce the artificial stabilization caused by basis-set sharing between the interacting fragments. In Equation 9, τ is the predicted recovery or desorption time in seconds; ν_0_ is the attempt frequency, taken as 10^12^ s^−1^; k_B_ is the Boltzmann Constant (1.380649 × 10^−23^ J.K^−1^; and T is the absolute temperature, taken as 298 K. In Equation 10, σ is the calculated electrical-conductivity-related quantity; A is the model prefactor; T is the absolute temperature in kelvin; Egap is the HOMO-LUMO energy gap in eV; k_B_ is the Boltzmann constant, 8.617333262 × 10^−5^ eV K^−1^, when Egap is used in eV (Hadi et al., 2024). Because the prefactor and electrode-dependent transport contributions are not explicitly determined, σ is used only for relative comparisons and should not be interpreted as an absolute conductivity.

Natural Population Analysis (NPA) and Mulliken population analysis were performed for the optimized isolated C_60_, BC_59_, and NC_59_ structures in the water phase at the B97D/6-31G(d) level to quantify substitution-induced changes in the atom-resolved charge distribution. NPA charges were used as the principal population descriptor, whereas Mulliken charges were included as a complementary scheme for assessing whether the qualitative charge-separation pattern was preserved. For BC_59_ and NC_59_, atom 60 corresponds to the substitutional B or N atom, respectively, while atoms C4, C13, and C_59_ are the three carbon atoms directly bonded to the heteroatom.

Quantum Theory of Atoms in Molecules was employed to analyze the nature of interactions by examining electron density at bond critical points (BCPs), providing insight into bond strength and type (covalent vs. noncovalent). In parallel, noncovalent interaction (NCI), reduced density gradient (RDG), Electron Localization Function (ELF), and Localized Orbital Locator (LOL) analyses were used to visualize weak interactions such as van der Waals forces and hydrogen bonding through characteristic colored isosurfaces (Savin et al., 1997). Together, these methods provide complementary information about the intermolecular interactions governing the calculated adsorption behavior and adsorption-induced electronic response.

All reported quantities were obtained from molecular models and approximate computational methods. The calculations do not explicitly represent electrode contacts, surface defects, finite-temperature molecular motion, explicit solvent molecules, competitive adsorption, or other experimental sources of variability. Consequently, the calculated adsorption energies, recovery times, and conductivity-related quantities should be interpreted primarily as comparative theoretical descriptors rather than direct laboratory observables. Experimental synthesis, structural characterization, and electrochemical measurements are required to evaluate the practical applicability of the proposed materials.

## 3 Results

For improved structural flow, the Results section is organized into dedicated subsections covering structural characterization and adsorption-configuration selection, reactivity descriptors and electronic-structure analysis, dipole moment, adsorption and predicted desorption behavior, NBO analysis, NCI/RDG analysis, ELF/LOL analysis, and QTAIM analysis.

### 3.1 Structural characterization and adsorption-configuration selection

Bond lengths and bond angles were analyzed to determine how substitutional B and N doping modifies the local geometry of the C_60_ cage. Heteroatom substitution breaks the symmetry of pristine C_60_ and may alter the local bonding environment and electron distribution (Shirley et al., 1995). The selected geometrical parameters are presented in Table 1.

**TABLE 1:** Selected bond lengths (Å) and bond angles (°) of the investigated structures calculated using the B97D and ωB97X-D functionals.

B97D					
Structure	Bond lengths			Bond angles	
​	​	Theoretical	Experimental (David et al., 1991)	​	​
C_60_	C1-C4	1.45	1.40 to 1.45	C1-C4-C2	119.98
	C2-C4	1.40		C1-C4-C3	107.99
	C3-C4	1.45		C2-C4-C3	119.99
BC_59_	C1-B	1.53	---------	C1-B-C2	118.46
	C2-B	1.55	---------	C1-B-C3	118.46
	C3-B	1.55	---------	C2-B-C3	106.26
NC_59_	C1-N	1.40	---------	C1-N-C2	119.22
	C2-N	1.42	---------	C1-N-C3	119.22
	C3-N	1.42	---------	C2-N-C3	107.41
ωB97XD					
C_60_	C1-C4	1.449	1.40 to 1.45	C1-C4-C2	120.02
	C2-C4	1.385		C1-C4-C3	108.00
	C3-C4	1.449		C2-C4-C3	119.97
BC_59_	C1-B	1.527	---------	C1-B-C2	118.57
	C2-B	1.546	---------	C1-B-C3	118.57
	C3-B	1.546	---------	C2-B-C3	106.29
NC_59_	C1-N	1.417	---------	C1-N-C2	106.86
	C2-N	1.417	---------	C1-N-C3	118.28
	C3-N	1.407	---------	C2-N-C3	118.28

A comparative analysis of the geometric parameters obtained for pristine and doped fullerenes reveals close agreement between the structural parameters predicted by the B97D and ωB97XD functionals, with both functionals capturing the same fundamental structural trends upon doping. For pristine C_60_, the two functionals predict closely comparable geometrical parameters, with both clearly distinguishing the two bond types characteristic of fullerene symmetry. B97D calculates the longer bonds at 1.45 Å and the shorter bonds at 1.40 Å, while ωB97XD yields nearly identical values of 1.449 Å and 1.385 Å, respectively. The bond angles are also highly consistent, both reporting values very close to the ideal 108° and 120°. This consistency extends to the boron-doped system, BC_59_. Both methods capture the significant bond elongation expected from boron’s larger atomic radius, with B97D reporting bonds of 1.53–1.55 Å and ωB97XD reporting slightly shorter but analogous values of 1.527–1.546 Å. The angular parameters predicted for BC_59_ are also in close agreement, showing the same distortion pattern with inner angles near 106.3° and outer angles near 118.5°. For the NC_59_ structure, both methods agree that bond lengths remain closer to pristine values than in the boron case. B97D places an angle of 107.41 degrees between C2-N-C3, while ωB97XD places an angle of 106.86 degrees between C1-N-C2. Overall, the results obtained with B97D and ωB97XD are in very good qualitative agreement. Both methods consistently show that boron doping leads to pronounced bond elongation and angular distortion, while nitrogen doping causes only minor structural changes and maintains the conjugated framework of C_60_. The close agreement between the qualitative results of the computational methods supports the robustness of the qualitative geometrical trends.

To determine whether the geometrical changes are accompanied by localized electron-density redistribution, the NPA and Mulliken atomic charges of the isolated C_60_, BC_59_, and NC_59_ cages were examined. The selected results are presented in Table 2.

**TABLE 2:** Selected NPA and Mulliken atomic charges for pristine C_60_ and for the substitutional heteroatom and its three directly bonded carbon neighbors in BC_59_ and NC_59_, calculated at the B97D/6-31G(d) level in water.

Structure	Atom/site	Relationship to substitution site	NPA charge (e)	Mulliken charge (e)
C_60_	All C atoms	Pristine reference	−0.00011 to +0.00015	−0.000334 to +0.000285
BC_59_	B_60_	Substitution atom	+0.69701	+0.153406
BC_59_	C_4_	Directly bonded to B_60_	−0.29055	−0.069721
BC_59_	C_13_	Directly bonded to B_60_	−0.29055	−0.069721
BC_59_	C_59_	Directly bonded to B_60_	−0.27164	−0.031350
NC_59_	N_60_	Substitution atom	−0.32780	−0.602024
NC_59_	C_4_	Directly bonded to N_60_	+0.22061	+0.256007
NC_59_	C_13_	Directly bonded to N_60_	+0.22061	+0.256007
NC_59_	C_59_	Directly bonded to N_60_	+0.20800	+0.237252

The atom-resolved population results provide quantitative evidence that heteroatom substitution disrupts the nearly uniform charge distribution of pristine C_60_. The carbon atoms of pristine C_60_ have nearly neutral NPA charges ranging from −0.00011 to +0.00015 e. In BC_59_, the substitutional B atom carries a positive NPA charge of +0.69701 e, whereas its three directly bonded carbon atoms, C4, C13, and C_59_, carry negative charges of −0.29055, −0.29055, and −0.27164 e, respectively. This charge pattern identifies the B atom as a locally electron-deficient center surrounded by electron-enriched neighboring carbon atoms.

An opposite charge-separation pattern is predicted for NC_59_. The substitutional N atom carries a negative NPA charge of −0.32780 e, while C4, C13, and C_59_ carry positive charges of +0.22061, +0.22061, and +0.20800 e, respectively. Mulliken population analysis predicts the same qualitative behavior, with a positively charged B center and negatively charged neighboring carbon atoms in BC_59_, and a negatively charged N center surrounded by positively charged carbon atoms in NC_59_. Therefore, both population schemes indicate that the strongest substitution-induced charge redistribution is localized at the heteroatom and the three directly bonded neighboring carbon atoms.

These electronic changes are accompanied by measurable geometrical and Frontier-orbital modifications. Compared with the C-C bond lengths of 1.40–1.45 Å in pristine C_60_, the B-C bonds in BC_59_ increase to 1.53–1.55 Å, whereas the N-C bonds in NC_59_ remain within approximately 1.40–1.42 Å. The accompanying changes in the local bond angles and the activation of additional calculated IR modes are consistent with a reduction in the high I_h_ symmetry of pristine C_60_ following substitution. At the B97D level, the HOMO-LUMO energy gap also decreases from 1.67 eV for C_60_ to 1.35 eV for BC_59_ and 0.38 eV for NC_59_. The ωB97XD calculations predict the same qualitative order, with corresponding values of 6.05, 5.72, and 4.31 eV. Thus, the combined geometrical, vibrational, atom-resolved charge, and Frontier-orbital results provide numerical support for substitution-induced modification of the local structure and electronic distribution of the fullerene cage.

To understand the vibrational distribution behavior as well as the bonding characteristics of the atoms, computational studies were performed on the IR spectra of each structure. Theoretical calculations of the IR spectra can be compared with experimental IR spectra and hence provide a theoretical description of the vibrational characteristics of the investigated structures (Mauron et al., 2012). In this research project, we calculated the IR spectra of C_60_ and its boron- and nitrogen-doped analogs, and also compared the calculated IR spectra of C_60_ with the experimentally measured IR spectra to determine how representative the computational method was in providing the correct IR spectra for C_60_.

For pristine C_60_, the calculated spectrum reproduces the characteristic vibrational pattern expected for a highly symmetric icosahedral (Ih) molecule. Only a limited number of IR-active modes are observed due to symmetry restrictions. The theoretical spectrum displays the main absorption bands in the mid-IR region, particularly around ∼1,400–1,500 cm^−1^ and ∼500–600 cm^−1^, which correspond to the well-known T1u vibrational modes of C_60_ associated with C=C stretching and cage deformation vibrations (Figure 1B).

Stability: Cohesive energy (E_Coh_) represents the energy required to separate a structure into its constituent isolated atoms and can be used as a comparative measure of the average internal bonding strength of a nanostructure. Previous DFT investigations of substitutionally doped C_59_X heterofullerenes, including BC_59_ and NC_59_, have similarly employed cohesive-energy calculations to evaluate how heteroatom substitution affects the energetic stability of the fullerene cage (Bai et al., 2013; Philipsen and Baerends, 1996). Accordingly, E_Coh_ is used in the present study as a first-order comparative energetic descriptor for determining whether B or N substitution substantially weakens the internal bonding network of C_60_. However, E_Coh_ alone should not be interpreted as definitive evidence of mechanical integrity, kinetic stability, experimental synthesizability, or long-term stability under operating conditions.

Under the sign convention adopted in the present work, a more negative E_Coh_ value corresponds to stronger average bonding between the constituent atoms. Pristine C_60_ exhibits an E_Coh_ value of −197.3 kcal.mol^−1^ atom^−1^, whereas the corresponding values for BC_59_ and NC_59_ are −195.9 and −196.1 kcal.mol^−1^ atom^−1^, respectively. Thus, both substitutionally doped structures exhibit slightly less negative E_Coh_ values than pristine C_60_, indicating a modest reduction in their intrinsic cohesive stability (Figure 2A). This small change in E_Coh_ accompanies the local geometrical distortion and atom-resolved charge separation quantified in Table 2. In particular, the positive charge on B and negative charges on its adjacent carbon atoms in BC_59_, together with the opposite charge-separation pattern in NC_59_, demonstrate that heteroatom substitution modifies the local electronic environment. However, the cohesive-energy results alone do not establish a direct causal relationship between charge redistribution and cohesive stability.

**FIGURE 2:**
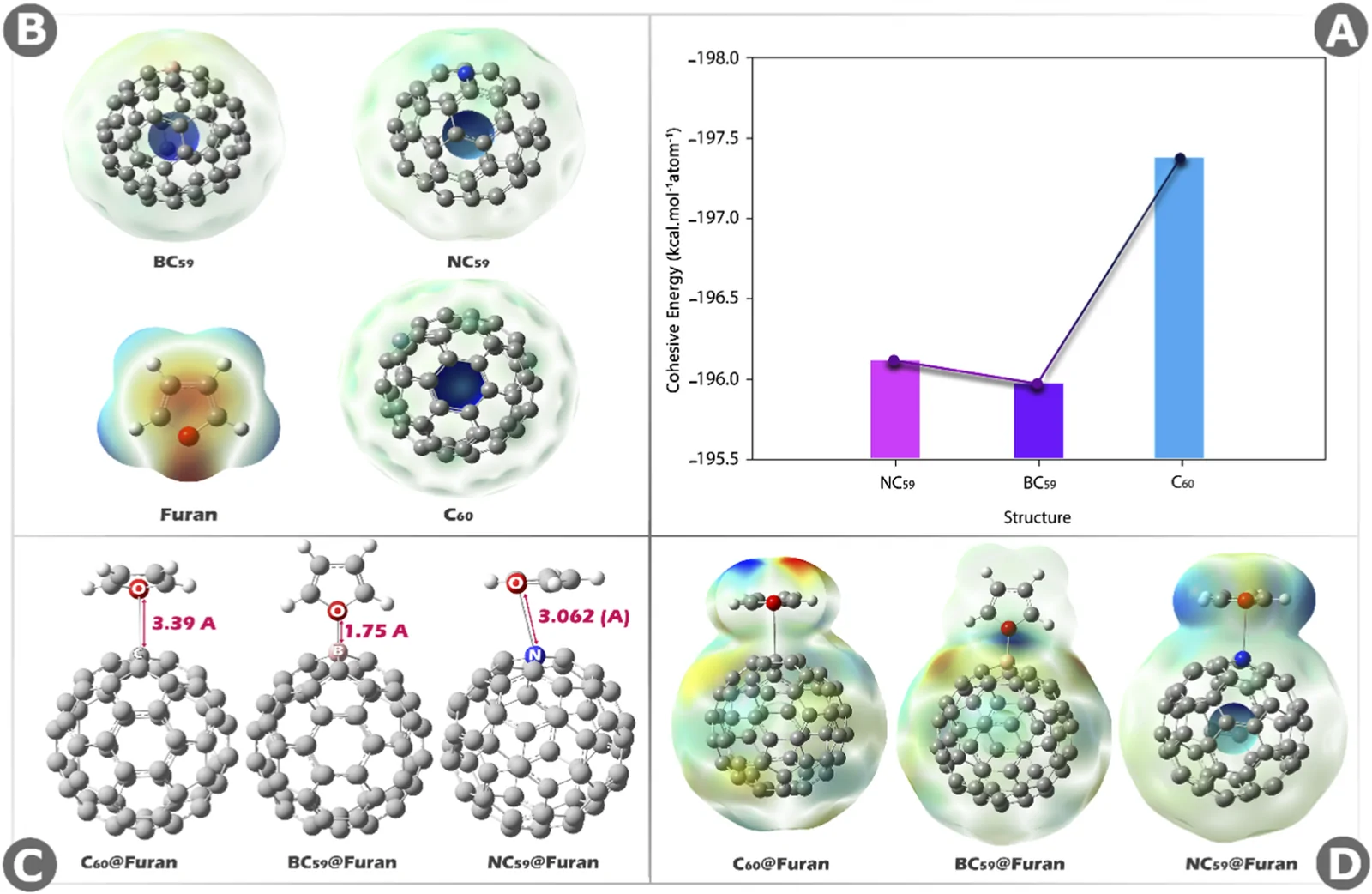
**(A)** Cohesive energy for pristine C_60_ fullerene and its doped forms (NC_59_, BC_59_). **(B)** MEP maps for furan, pristine C_60_, BC_59_, and NC_59_. **(C)** Bond lengths of optimized geometries C_60_@Furan, BC_59_@Furan, and NC_59_@Furan. **(D)** MEP contours for each of the designed complexes.

MEP maps visualize electrostatic potential across a molecule’s electron-density surface, distinguishing electron-rich red regions, electron-deficient blue regions, and intermediate green-to-yellow areas (Lakshminarayanan et al., 2021). These maps help identify likely interaction sites and guide the design of plausible initial adsorption configurations for furan with C_60_, BC_59_, and NC_59_, as shown in Figures 2B,D.

The MEP map of furan shows a pronounced negative-potential region around its oxygen atom, consistent with its lone-pair electron density. The starting adsorption sites were selected by considering the MEP surfaces together with the atom-resolved NPA charges in Table 2. The three sites were topologically matched: the exposed carbon position used as the reference site in pristine C_60_ corresponds to the cage position occupied by B or N in the doped models. This choice retains comparable local curvature and accessibility while allowing the effect of substitutional atom identity to be examined. Because pristine C_60_ has an approximately uniform potential and nearly neutral carbon atoms, the selected carbon position provides a representative pristine-cage reference. In BC_59_, the substitutional B atom carries a positive NPA charge of +0.69701 e and therefore constitutes the most electron-deficient local center; the B-centered region was accordingly chosen to test a direct interaction with the electron-rich oxygen end of furan. The rationale for NC_59_ was different. The substitutional N atom is electron-rich (−0.32780 e), whereas its three directly bonded carbon atoms are positively charged (+0.20800 to +0.22061 e). The N-centered doped region was nevertheless sampled as a controlled dopant-site configuration to determine how the reversed dopant polarity and polarization of the neighboring carbon atoms affect the preferred approach of furan. Thus, the NC_59_ site was not selected based on the assumption that N is an electron-deficient center.

At each selected surface region, two chemically distinct orientations of furan were constructed to reduce dependence on a single initial geometry. In the oxygen-directed orientation, the furan O atom was pointed toward the representative carbon region of C_60_ or the substitution-centered region of BC_59_ and NC_59_. This arrangement samples a possible interaction involving the oxygen lone pair and allows the effects of B and N substitution to be compared at analogous cage sites without presuming that the two dopants have the same polarity. In the second orientation, furan was placed edge-on, with an alpha-carbon/C-H region directed toward the same surface region, to probe a contrasting motif involving C-H···surface contacts, dispersion, and polarization-assisted interactions. Keeping the surface region approximately constant while changing the molecular approach helps distinguish the effect of orientation from that of adsorption-site identity. These two starting geometries represent chemically motivated limiting motifs rather than an exhaustive global conformational search (Supplementary Figure S3 in the Supplementary Data).

Each of the two initial configurations was independently subjected to full, unconstrained geometry optimization at the same computational level. The resulting optimized structures were then compared based on their total energies, adsorption energies, final interaction motifs, and intermolecular distances. The configuration with the lowest calculated energy among the two sampled orientations was selected for the subsequent electronic, adsorption, NBO, NCI, ELF/LOL, and QTAIM analyses. The selected optimized geometries of C_60_@Furan, BC_59_@Furan, and NC_59_@Furan are shown in Figure 2C.

The optimized complexes exhibit the shortest intermolecular contact distances of 1.75, 3.06, and 3.39 Å for BC_59_@Furan, NC_59_@Furan, and C_60_@Furan, respectively. However, the stronger electronic interaction predicted for BC59@Furan is not inferred from the interaction distance alone. At the B97D level, furan adsorption decreases the HOMO-LUMO energy gap of BC59 from 1.35 to 1.09 eV, corresponding to an adsorption-induced reduction of 0.26 eV. By comparison, the corresponding reductions are only 0.14 eV for C_60_, from 1.67 to 1.53 eV, and 0.03 eV for NC_59_, from 0.38 to 0.35 eV. The ωB97XD calculations predict the same qualitative behavior, with the largest gap reduction obtained for BC_59_, 0.37 eV, compared with 0.08 eV for both C_60_ and NC_59_ (Table 3). The NBO results provide additional evidence of enhanced orbital delocalization within the BC_59_@Furan complex. The selected π→π* interaction has an E^(2)^ stabilization energy of 14.16 kcal.mol^−1^ for BC_59_@Furan, compared with 7.22 and 7.30 kcal.mol^−1^ for C_60_@Furan and NC59@Furan, respectively. Similarly, the selected LP→π* stabilization energy is 61.81 kcal.mol^−1^ for BC_59_@Furan, substantially higher than the corresponding values of 32.40 and 27.96 kcal.mol^−1^ for C60@Furan and NC_59_@Furan (Table 4). Furthermore, the QTAIM analysis shows substantially greater electron-density accumulation at the intermolecular bond critical point of BC59@Furan, with ρ(r) values of 0.067 and 0.076 a.u. at the B97D and ωB97XD levels, respectively, compared with values of only 0.006–0.008 a.u. for C_60_@Furan and NC_59_@Furan (see Table 6). Collectively, these calculated electronic-structure descriptors support a more localized and electronically significant interaction at the doped site in BC_59_@Furan, whereas the longer contacts in C_60_@Furan and NC_59_@Furan are associated with weaker local electron-density accumulation. Accordingly, the 1.75 Å distance should be interpreted as a short calculated intermolecular contact consistent with enhanced electronic coupling and electron-density redistribution, rather than being used alone as direct proof of π-electron transfer.

**TABLE 3:** Frontier molecular orbital energies and reactivity descriptors of C_60_, doped fullerenes, and their complexes.

Structure	LUMO (eV)	HOMO (eV)	E_gap_ (eV)	η (eV)	μ (eV)	S (eV^-1^)	${\Delta N}_{\max}$ (e)	ECT (e)
B97D								
C_60_	−3.58	−5.25	1.67	0.83	−4.41	0.59	5.28	---------
BC_59_	−3.58	−4.93	1.35	0.67	−4.25	0.74	6.30	---------
NC_59_	−3.64	−4.02	0.38	0.19	−3.83	2.63	20.15	---------
C_60_@Furan	−3.54	−5.07	1.53	0.76	−4.30	0.65	5.62	−0.34
BC_59_@Furan	−3.43	−4.52	1.09	0.54	−3.97	0.91	7.29	−0.98
NC_59_@Furan	−3.61	−3.96	0.35	0.17	−3.78	2.85	21.62	−1.47
ωB97XD								
C_60_	−1.67	−7.72	6.05	3.025	−4.69	0.16	1.55	---------
BC_59_	−1.68	−7.4	5.72	2.86	−4.54	0.17	1.58	---------
NC_59_	−1.72	−6.03	4.31	2.155	−3.87	0.23	1.79	---------
C_60_@Furan	−1.66	−7.63	5.97	2.985	−4.64	0.16	1.55	−0.004
BC_59_@Furan	−1.55	−6.9	5.35	2.675	−4.22	0.18	1.57	−0.007
NC_59_@Furan	−1.71	−5.94	4.23	2.115	−3.82	0.23	1.80	−0.010

At the B97D level, the HOMO-LUMO, energy gap decreases from 1.67 eV for pristine C_60_ to 1.35 eV for BC_59_ and 0.38 eV for NC_59_. The corresponding chemical-hardness values decrease from 0.83 eV to 0.67 and 0.19 eV, while the chemical-softness values increase from 0.59 eV^-1^, to 0.74 and 2.63 eV^-1^, respectively. These results indicate that substitutional doping increases the calculated electronic responsiveness of the C_60_ cage, particularly for NC_59_.

**TABLE 4:** Calculated values of σ, Eads and τ for C_60_, BC_59_ and NC_59_ in the presence and absence of Furan.

Structure	Eads (kcal.mol^−1^)	*τ* (s)	Log_10_(*τ*)	(*σ*) (A.m)
B97D				
C_60_	----------	----------	----------	2.20 × 10^9^
BC_59_	----------	----------	----------	2.35 × 10^9^
NC_59_	----------	----------	----------	2.86 × 10^9^
C_60_@Furan	−24.36	7.23 × 10^5^	5.86	2.27 × 10^9^
BC_59_@Furan	−34.61	4.46 × 10^13^	13.65	2.48 × 10^9^
NC_59_@Furan	−28.54	8.45 × 10^8^	8.93	2.88 × 10^9^
ωB97XD				
C_60_	----------	----------	----------	9.10 × 10^8^
BC_59_	----------	----------	----------	9.74 × 10^8^
NC_59_	----------	----------	----------	1.29 × 10^9^
C_60_@Furan	−21.86	1.07 × 10^4^	4.03	9.26 × 10^8^
BC_59_@Furan	−49.44	1.26 × 10^24^	24.10	1.05 × 10^9^
NC_59_@Furan	−27.08	7.24 × 10^7^	7.86	1.31 × 10^9^

**TABLE 5:** Calculated values of NBO analysis using the B97D functional for the studied complexes.

Complex	Donor(i)	Type	acceptor(j)	Type	E^(2)^ kcal.mol^−1^	εj-εi (a.u.)	F(i,j) a.u.
B97D							
C_60_@Furan	C1-C2	σ	C1-C4	$\sigma^{*}$	1.16	1.09	0.045
	C37-C38	π	C28-C29	$\pi^{*}$	7.22	0.25	0.054
	C_59_	LP (1)	C10-C11	$\pi^{*}$	32.40	0.13	0.091
BC_59_@Furan	C1-C2	σ	C4-C15	$\sigma^{*}$	2.80	1.00	0.047
	C1-C4	π	C15-C16	$\pi^{*}$	14.16	0.26	0.055
	C_59_	LP (1)	C2-C3	$\pi^{*}$	61.81	0.12	0.087
NC_59_@Furan	C1-C2	σ	C3-C9	$\sigma^{*}$	1.25	1.00	0.045
	C2-C3	π	C7-C8	$\pi^{*}$	7.30	0.26	0.056
	C_59_	LP (1)	C10-C11	$\pi^{*}$	27.96	0.15	0.088

**TABLE 6:** QTAIM parameters at the bond critical points (BCPs) for C_60_@Furan, NC_59_@Furan, and BC_59_@Furan complexes. All values are in atomic units.

Structure	ρ(r)	G(r)	V(r)	∇2ρ(r)	Hb
B97D					
C_60_@Furan	0.007	0.004	-0.003	0.020	0.001
BC_59_@Furan	0.067	0.089	-0.121	0.204	-0.032
NC_59_@Furan	0.008	0.0068	-0.006	0.028	0.0005
ωB97XD					
C_60_@Furan	0.006	0.003	-0.003	0.020	0.001
BC_59_@Furan	0.076	0.129	-0.166	0.372	-0.036
NC_59_@Furan	0.007	0.006	-0.006	0.024	0.0005

### 3.2 Reactivity descriptors and electronic-structure analysis

Reactivity descriptors were used to evaluate the electronic response of C_60_, BC_59_, and NC_59_ toward furan adsorption. A smaller energy gap, lower hardness, and higher softness indicate a more significant electronic response (Harbola et al., 1991; Makov, 1995; Kaya and Putz, 2022). Chemical potential describes the tendency for electron flow, ΔNmax estimates charge-transfer capacity, and ECT indicates its direction: negative values represent electron transfer from furan to the fullerene system, whereas positive values indicate the reverse. The calculated results are presented in Table 3.

The chemical potential (μ) becomes slightly less negative with doping, shifting from −4.54 in C_60_ to −4.25 in BC_59_ and -4.49 in NC_59_, implying a greater tendency to donate electrons. The maximum charge transfer capacity (ΔN_max_) also increases in both doped structures (6.30 for BC_59_ and 6.37 for NC_59_ compared to 5.40 in C_60_), suggesting improved capability for electron exchange. Collectively, these results indicate that doping enhances the reactivity and charge-transfer potential of C_60_, with both BC_59_ and NC_59_ offering improved properties over the pristine form.

After interaction with furan, changes in Egap, η, S, μ, and ΔNmax provide a comparative description of adsorption-induced electronic perturbation, whereas ECT is used only to identify the predicted direction of charge transfer. For C_60_@Furan, Egap decreases from 1.67 to 1.53 eV, η decreases from 0.83 to 0.76 eV, and softness increases from 0.59 to 0.65 eV^-1^. The negative sign of ECT qualitatively indicates a predicted charge-transfer direction from furan to C_60_. For BC_59_@Furan, Egap decreases from 1.35 to 1.09 eV, η decreases from 0.67 to 0.54 eV, and softness increases from 0.74 to 0.91 eV^-1^. The negative ECT sign similarly indicates a predicted charge-transfer direction from furan to BC_59_. For NC_59_@Furan, Egap decreases slightly from 0.38 to 0.35 eV, η decreases from 0.19 to 0.17 eV, and softness increases from 2.63 to 2.85 eV^-1^. Its negative ECT sign indicates a predicted charge-transfer direction from furan to NC59. Thus, at the B97D level, all three complexes exhibit the same qualitative charge-transfer direction from furan toward the fullerene-based adsorbent. Differences in electronic response and interaction strength are evaluated using the adsorption-induced changes in the electronic descriptors together with the structural, NBO, NCI, and QTAIM results, rather than by comparing ECT magnitudes.

In addition to the B97D results, the electronic properties calculated using the ωB97XD functional provide further insight into the robustness of the predicted qualitative electronic-response trends. Here, the ωB97XD results are discussed as a trend validation (robustness) analysis. Owing to its inclusion of long-range exchange correction and empirical dispersion, ωB97XD generally produces larger absolute HOMO-LUMO gaps compared to B97D, while preserving meaningful relative trends (see Figure 3A). For pristine C_60_, ωB97XD yields HOMO and LUMO energies of −7.72 eV and −1.67 eV, respectively, corresponding to a gap of 6.05 eV. Upon boron doping (BC_59_), the gap decreases to 5.72 eV, while nitrogen doping (NC_59_) results in a more substantial reduction to 4.31 eV, indicating enhanced electronic reactivity upon heteroatom substitution. After furan adsorption, the energy gaps further decrease to 5.97 eV for C_60_@Furan, 5.35 eV for BC_59_@Furan, and 4.23 eV for NC_59_@Furan. Although the absolute gap values differ from those predicted by B97D, adsorption-induced electronic perturbations are clearly observed in all complexes. A similar trend is reflected in the chemical hardness (η) and softness (S) descriptors, where doping reduces hardness and increases softness relative to pristine C_60_, confirming improved electronic flexibility (See Figures 3C,D). At the ωB97XD level, the ECT values listed in Table 3 are negative for C_60_@Furan, BC_59_@Furan, and NC_59_@Furan. Only their signs are interpreted, indicating a predicted charge-transfer direction from furan toward each fullerene-based adsorbent. Their numerical magnitudes are not used to infer limited or extensive charge transfer and are not compared among the complexes. Therefore, the B97D and ωB97XD results show qualitative agreement regarding the predicted direction of charge transfer.

**FIGURE 3:**
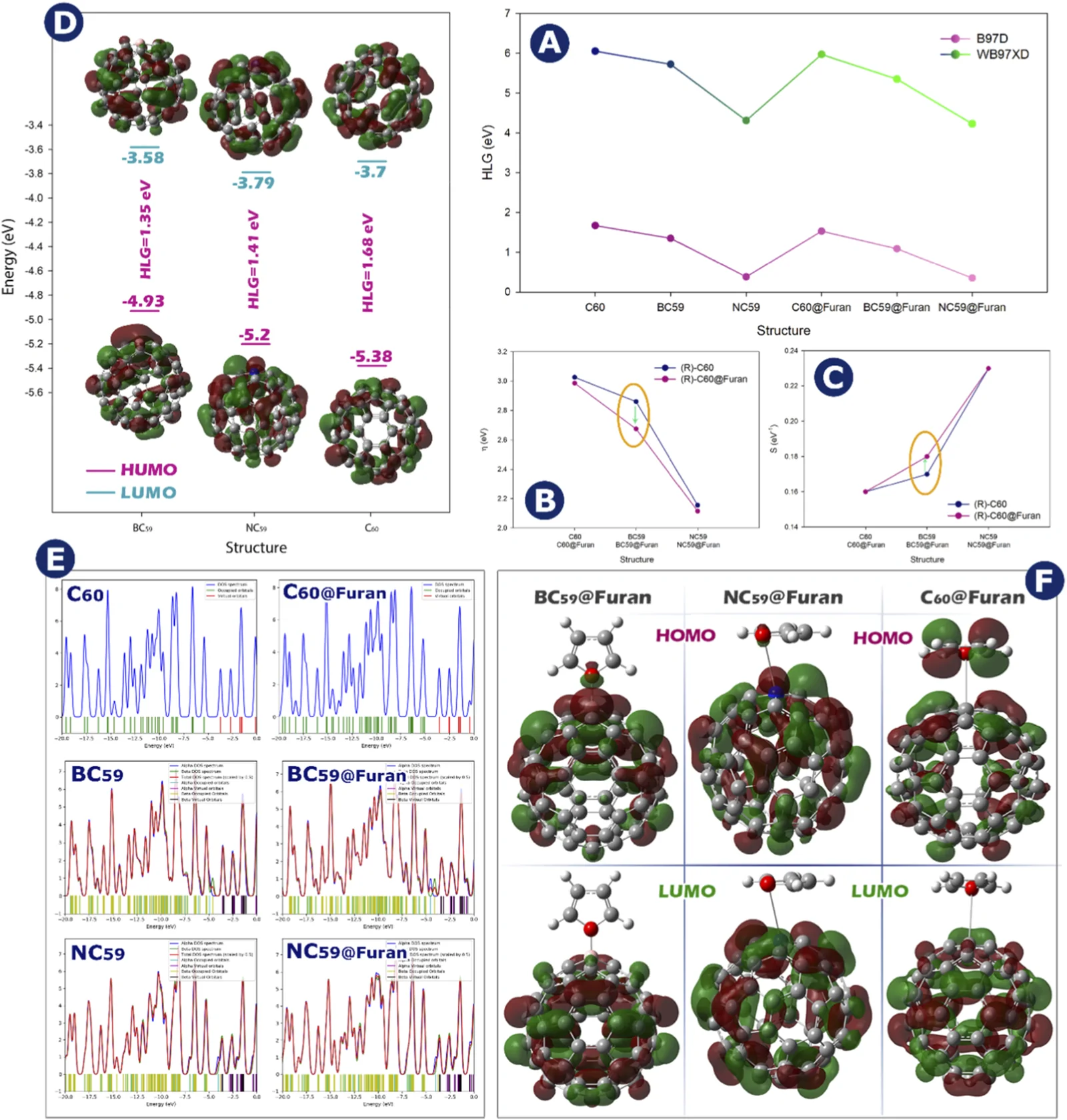
**(A)** Comparison of the HLG results calculated with two computational functionals B97D and ωB97XD. The trend of changes in **(B)** chemical hardness and **(C)** chemical softness in the presence and absence of furan. **(D)** Comparison of HOMO/LUMO energy and energy gap for C_60_, BC_59_, and NC_59_ (green surfaces represent positive phases and red surfaces represent negative phases of molecular orbitals). **(E)** DOS contours for pristine C_60_ and its doped forms in the presence/absence of furan. **(F)** HOMO and LUMO orbital distributions for BC_59_@Furan, NC_59_@Furan, and C_60_@Furan. Green and red isosurfaces represent the positive and negative phases of the molecular orbitals, respectively.

The spatial distributions of the HOMO and LUMO provide qualitative information about the regions involved in electron donation and acceptance (Lu et al., 2003). In C_60_@Furan, the HOMO is distributed over much of the complex, whereas the LUMO is predominantly localized on the C_60_ cage, indicating that the cage is the principal electron-accepting region. In BC_59_@Furan and NC_59_@Furan, both Frontier orbitals are mainly localized on the doped fullerene moieties. These distributions suggest that B and N substitution modifies the location and degree of Frontier-orbital localization and may therefore influence the adsorption-induced electronic response shown in Figure 3F.

The density-of-states plots provide a graphical representation of the calculated electronic states and the separation between the occupied and unoccupied regions (Huang et al., 2017; Toriyama et al., 2022). The HOMO-LUMO separations visible in Figure 3E are consistent with the corresponding values listed in Table 3. The changes in peak position and intensity following furan adsorption indicate adsorption-induced perturbation of the electronic structures, particularly for BC_59_. These observations support the relative electronic-response trends but do not independently establish experimental conductivity or sensing performance.

Total density of states and projected density of states analyses were used to examine the electronic-state distributions before and after furan adsorption. TDOS represents the overall distribution of calculated states, whereas PDOS separates the contributions associated with selected atomic or orbital components. Adsorption-induced peak shifts, broadening, or changes near the reference energy may indicate electronic perturbation and orbital mixing. However, these calculated molecular-state distributions should not be interpreted as direct measurements of the electronic structure of a fabricated electrode (see Figure 4).

**FIGURE 4:**
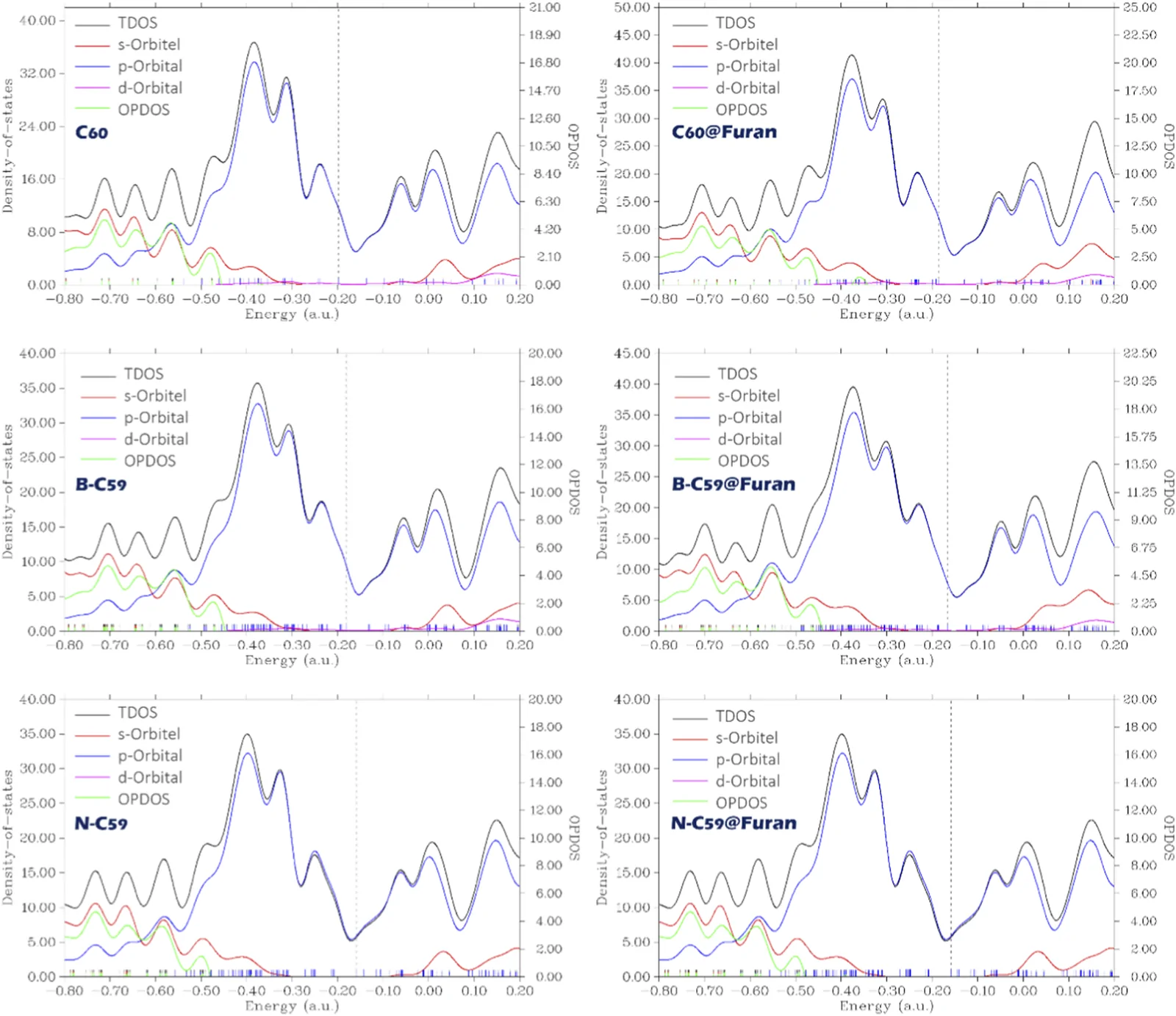
Total density of states (TDOS) and projected density of states (PDOS) for C_60_, BC_59_, and NC_59_ before and after furan adsorption. The TDOS (black line) represents the overall electronic states, while PDOS curves show the contributions of s, p, and d orbitals. The vertical dashed line indicates the Fermi level.

For pristine C_60_, the calculated states are dominated by p-orbital contributions, consistent with its π-conjugated carbon framework. Furan adsorption produces only limited changes in the TDOS and PDOS profiles of C_60_, suggesting a comparatively weak electronic perturbation. Boron substitution modifies the state distribution of BC_59_ before adsorption. Following furan adsorption, BC_59_@Furan exhibits more pronounced peak shifts, broadening, and redistribution near the Frontier-orbital region than C_60_@Furan. These changes are consistent with the stronger adsorption-induced electronic perturbation predicted by the energy-gap, NBO, and QTAIM analyses. NC_59_ has a substantially smaller calculated energy gap than pristine C_60_ even before adsorption. However, furan adsorption produces only a small additional reduction in its energy gap and moderate changes in its DOS profile. Therefore, the results suggest that NC_59_ is intrinsically electronically responsive, whereas BC_59_ undergoes the largest adsorption-induced change among the investigated systems.

### 3.3 Dipole moment

Dipole moment was examined as a descriptor of molecular polarity and adsorption-induced charge redistribution. An increase in dipole moment after adsorption may indicate greater polarization of the complex and could influence its interaction with an external electric field or polar environment (Hait and Head-Gordon, 2018). However, dipole moment alone does not establish experimental signal strength, sensitivity, or solubility. The calculated results are presented in Figure 5 and Supplementary Figure S4 (In Supplementary Data).

**FIGURE 5:**
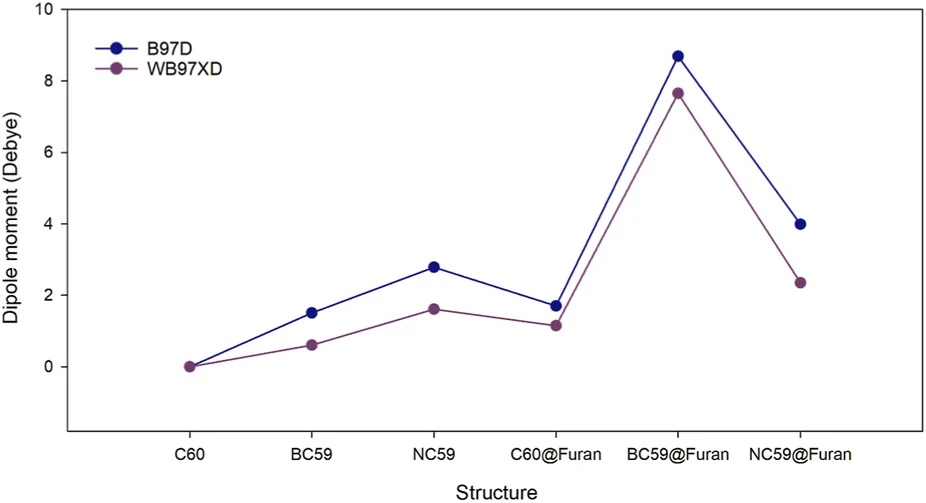
Calculated dipole moments of the investigated fullerene systems before and after furan adsorption.

Although the B97D and ωB97X-D calculations produce different absolute dipole moments, they predict comparable qualitative trends (see Figure 5). Pristine C_60_ has a dipole moment of 0.0 D because of its high molecular symmetry. Substitution with B or N breaks this symmetry and increases the dipole moment. Furan adsorption causes a further increase, with the largest adsorption-induced change predicted for BC_59_@Furan (see Supplementary Figure S4 in Supplementary Data). This result suggests greater charge redistribution in the boron-substituted complex and is consistent with the complementary electronic-structure analyses.

### 3.4 Adsorption energy, predicted desorption behavior, and conductivity-related response

Adsorption energy, predicted desorption time, and the conductivity-related quantity were used as comparative descriptors of adsorption strength, regenerability, and adsorption-induced electronic response. Moderately favorable adsorption is generally desirable for reusable sensing because it permits analyte binding without preventing subsequent desorption. By contrast, very strong adsorption and long predicted desorption times may be more appropriate for permanent adsorption or disposable detection. For an experimentally useful conductometric platform, the adsorption-induced change in electrical response is more relevant than a high calculated conductivity value alone (Zandi et al., 2026). The calculated quantities reported in Table 4 should therefore be interpreted as theoretical comparisons rather than direct measurements of device performance.

Table 4 summarizes the calculated Eads, τ, and σ of C_60_, BC_59_, and NC_59_ in the absence and presence of Furan using the B97D and ωB97XD, functionals. At the B97D level, the calculated adsorption-strength order is BC_59_@Furan > NC_59_@Furan > C_60_@Furan, with adsorption energies of −34.61, −28.54, and −24.36 kcal.mol^−1^, respectively. The ωB97X-D functional predicts the same qualitative order, although it gives a substantially more negative value for BC_59_@Furan. This difference indicates a functional dependence in the absolute adsorption energies, although qualitative predictions show a similar trend. At both levels of theory, the calculated adsorption-strength order is BC_59_@Furan > NC_59_@Furan > C_60_@Furan. although the absolute adsorption energies differ between the two functionals, both computational methods suggest BC_59_@Furan as a candidate complex for further experimental evaluation (Figure 6A).

**FIGURE 6:**
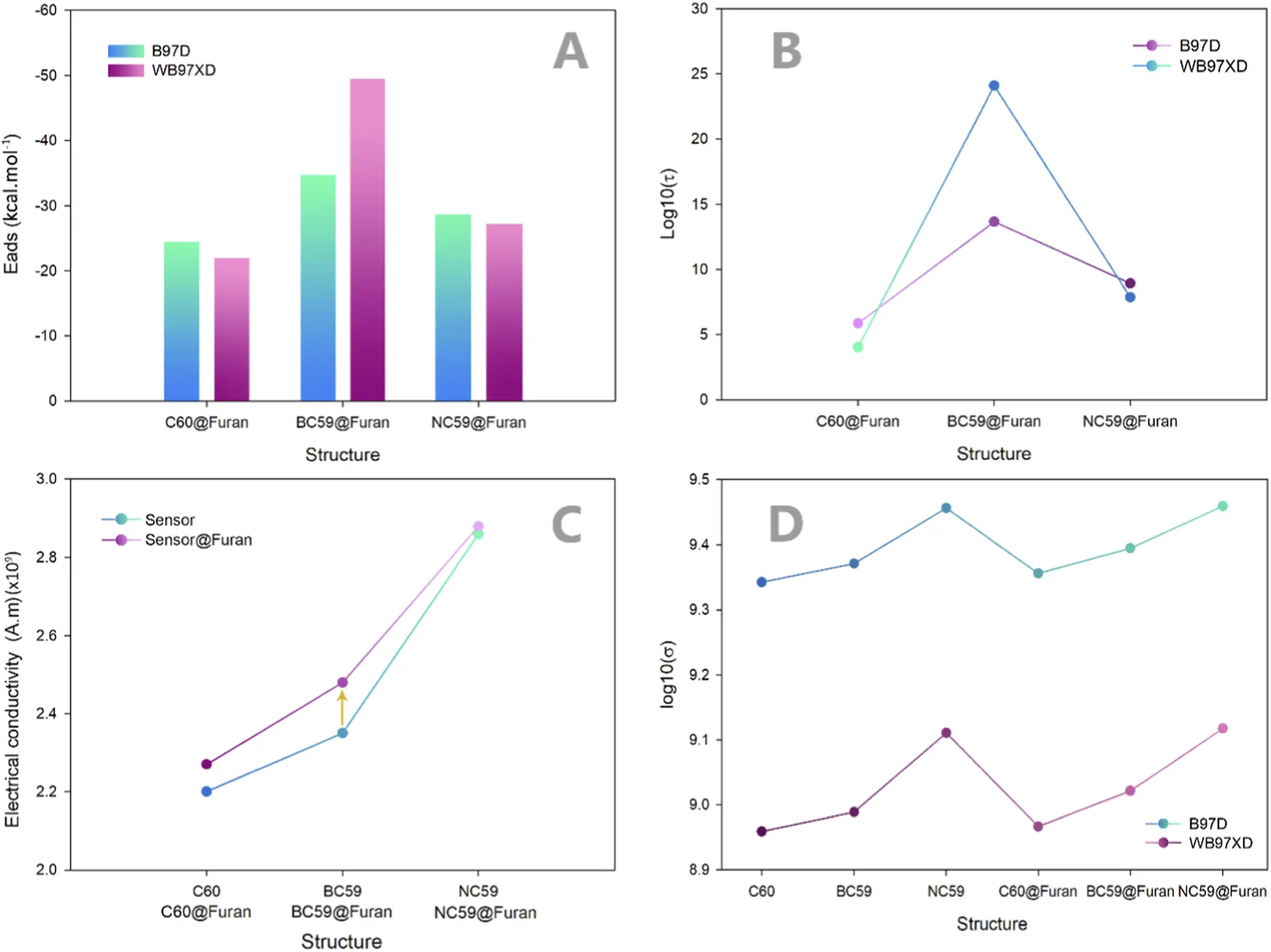
**(A)** Adsorption energies calculated using the B97D and ωB97X-D functionals. **(B)** Log_10_(τ) values for furan adsorption on C_60_, BC_59_, and NC_59_. **(C)** Adsorption-induced changes in the conductivity-related quantity calculated using B97D. **(D)** Logarithmic comparison of the conductivity-related quantities calculated using B97D and ωB97X-D.

The calculated recovery times, when correctly evaluated using the Arrhenius-type equation (Equation 9), reveal important physical limitations for sensing applications. For both functionals, C60@Furan exhibits the shortest predicted recovery times (τ ≈ 7.23 × 10^5^ s ≈ 8.4 days with B97D; τ ≈ 1.07 × 10^4^ s ≈ 3 h with ωB97XD), followed by NC59@Furan (τ ≈ 8.45 × 10^8^ s (B97D); τ ≈ 7.24 × 10^7^ s (ωB97XD)), whereas BC59@Furan shows exceptionally long τ values (τ ≈ 4.46 × 10^13^ s ≈ 1.4 million years with B97D; τ ≈ 1.26 × 10^24^ s ≈ 4 × 10^16^ years with ωB97XD). In particular, ωB97X-D, which includes long-range exchange and empirical dispersion interactions, predicts an essentially irreversible interaction for BC_59_@Furan, consistent with its very strong calculated adsorption energy (−49.44 kcal.mol^−1^). Such exceptionally long predicted desorption times are consistent with effectively irreversible adsorption under the assumptions of the model and are incompatible with rapid room-temperature regeneration. Under the assumptions of the present model, this result suggests that BC_59_ may be unsuitable for rapidly reusable sensing applications but could be considered a candidate permanent adsorbent for further experimental evaluation.

The predicted desorption behavior of each investigated complex was compared using the Log_10_(τ) values calculated with the B97D and ωB97XD functionals. Overall, both computational functionals predict that the doped structures show distinct recovery characteristics compared to the original C60 complex, highlighting the dependence of desorption behavior on fullerene composition (Figure 6B).

Based on both computational methods, BC_59_@Furan consistently shows the longest predicted recovery time, indicating the strongest interaction and lowest tendency to desorb. C_60_@Furan shows the smallest predicted recovery, though even the shortest predicted desorption time (≈3 h with ωB97XD) is relatively long for practical sensing applications. NC_59_@Furan falls between the two, with recovery times on the order of years, indicating strong but not irreversible binding. The qualitative ranking (BC_59_ > NC_59_ > C_60_) is consistent across both functionals, though the absolute values differ significantly due to the different treatment of dispersion interactions. Critically, none of the systems exhibit the fast desorption (τ < 1s) typically required for real-time reversible sensing applications at room temperature. Therefore, within the assumptions of the present model, these structures are not predicted to be suitable for repeatedly regenerable sensing platforms and may be more appropriate for evaluation in single-use detection configurations. Although B97D and ωB97XD yield τ values with different absolute magnitudes, they derive the same qualitative ranking of recovery behavior for each system. This consistency supports the robustness of the relative sensing trend predicted by these computations. It should be noted that the study is solely theoretical; thus, the τ values found in this study should only be considered as qualitative indicators for comparing relative desorption/predicted recovery and not as empirical recovery times.

Furan adsorption also induces a slight increase in electrical conductivity for all systems. This enhancement is modest for pristine C_60_ but more pronounced for the doped fullerenes, especially NC_59_, which maintains the highest conductivity before and after adsorption in both computational schemes. The consistent increase in σ upon Furan adsorption suggests effective charge transfer and supports the potential of these materials for chemoreceptive sensing.

The conductivity-related quantities calculated for the isolated structures and their furan complexes are compared in Figures 6C,D. These plots illustrate relative adsorption-induced changes and should not be interpreted as experimentally measured conductivities. Among the investigated systems, BC_59_ shows the largest relative change in Log_10_σ following furan adsorption, suggesting a comparatively pronounced electronic response. Experimental transport measurements are required to determine whether this change would produce a measurable signal in a fabricated device.

Our results indicate that substitutional doping with boron or nitrogen generally enhances the intrinsic conductance of the fullerene framework relative to pristine C_60_. Notably, among the investigated structures, BC_59_ exhibits the largest relative change in Log_10_σ upon furan adsorption. These values are consistently greater than those observed for C_60_ and NC_59_, suggesting that the electron-deficient nature of the boron center may facilitate more effective charge-transfer interactions with the furan molecule (Figure 6C).

### 3.5 Natural bond orbital analysis

Natural Bond Orbital analysis was used to characterize donor–acceptor interactions and electron-density delocalization within the optimized complexes. The second-order perturbation stabilization energy, E^2^, provides a measure of the calculated interaction between an occupied donor NBO and an unoccupied acceptor NBO. Larger E^2^ values indicate stronger donor-acceptor delocalization for the selected orbital pair. These values can support interpretation of adsorption-induced polarization and orbital redistribution but should not be treated as direct measures of experimental sensitivity or electrical conductivity. E^2^ is calculated using Equation 11.

$$E^{2}={\Delta E}_{i,j}=q\frac{F^{2}\left(i,j\right)}{E_{j}-E_{i}}$$ (11)

where E^2^, also denoted ΔEij, is the second-order perturbation stabilization energy associated with electron-density donation from donor NBO i to acceptor NBO j, reported in kcal.mol^−1^; qi is the occupancy of donor orbital i, expressed in electrons; F (i,j) is the off-diagonal Fock-matrix element between donor orbital i and acceptor orbital j, expressed in atomic units (a.u.); εi and εj are the energies of the donor and acceptor NBOs, respectively, in a.u.; and i and j identify the donor and acceptor orbitals, respectively. LP denotes a lone-pair orbital; σ and π denote bonding orbitals; and σ∗ and π∗ denote the corresponding antibonding orbitals. The asterisk (∗) identifies an antibonding orbital (Hadi and Alshehri, 2025).

The selected NBO interactions in Table 5 are consistently stronger for BC_59_@Furan than for C_60_@Furan and NC_59_@Furan. The σ→σ*, π→π*, and LP→π* interactions in BC_59_@Furan have E^(2)^ values of 2.80, 14.16, and 61.81 kcal.mol^−1^, respectively. The corresponding values are lower for the other two complexes. These results suggest stronger calculated donor-acceptor delocalization and polarization within the boron-substituted complex.

### 3.6 Noncovalent-interaction and reduced-density-gradient analyses

Noncovalent interaction and reduced-density-gradient analyses were used to visualize weak intermolecular interactions in the optimized complexes. The quantity sign (λ_2_)ρ assists in distinguishing attractive regions, values near zero associated mainly with weak dispersion, and positive regions associated with steric repulsion. The corresponding NCI/RDG plots are presented in Figure 7 (Hanif et al., 2026).

**FIGURE 7:**
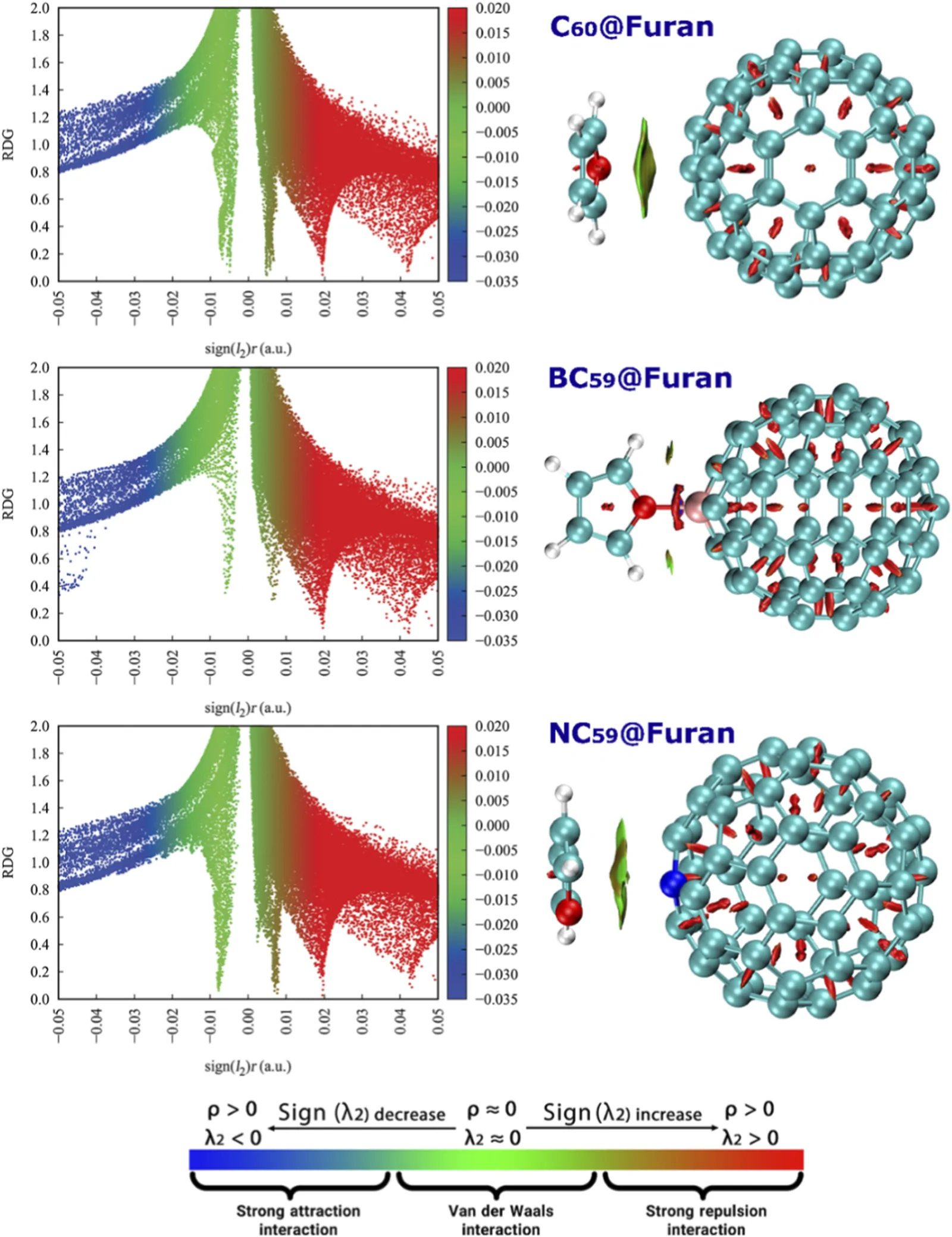
NCI/RDG contour for C_60_@Furan, BC_59_@Furan, and NC_59_@Furan.

Although C_60_@Furan contains peaks from attractive interactions, they are somewhat narrow, indicating weakly dispersed contacts in this compound. In comparison, NC_59_@Furan shows a slight broadening and intensification in the attractive region relative to C_60_@Furan, which is consistent with its moderately stronger calculated interaction. The apparent similarity between the NCI plots of C_60_@Furan and NC_59_@Furan is expected because both systems are governed predominantly by weak dispersion-dominated physisorption, leading to qualitatively similar NCI signatures. Their difference is mainly quantitative, with NC_59_@Furan showing only a slight broadening/intensification in the attractive (negative sign (λ_2_)ρ) region, consistent with its marginally stronger interaction relative to C_60_@Furan. However, BC_59_@Furan stands out with the most pronounced and extended peaks in the negative sign (λ_2_)ρ domain, indicating a significant enhancement in attractive interactions compared to the other systems. This response suggests that boron doping may increase the magnitude of dispersion forces and potentially introduce additional electrostatic contributions in the computational model. The broader and sharper peaks imply a stronger stabilization of the BC_59_@Furan complex, reflecting a tighter and more energetically favorable binding interface. These results highlight BC_59_@Furan as the system with the most substantial non-covalent interaction signature in the computational models, suggesting that boron substitution may amplify the host-guest affinity in fullerene-furan complexes.

### 3.7 Electron-localization-function and localized-orbital-locator analyses

Electron Localization Function and Localized Orbital Locator maps were used to examine the spatial distribution of localized and delocalized electron density. Regions with values approaching one indicate strong electron localization, whereas values approaching 0 indicate weak localization or low electron density (Bandyopadhyay et al., 2020). These maps provide qualitative information about polarization and localization near the adsorption interface but do not by themselves prove covalent-bond formation or quantify charge transfer (see Figure 8).

**FIGURE 8:**
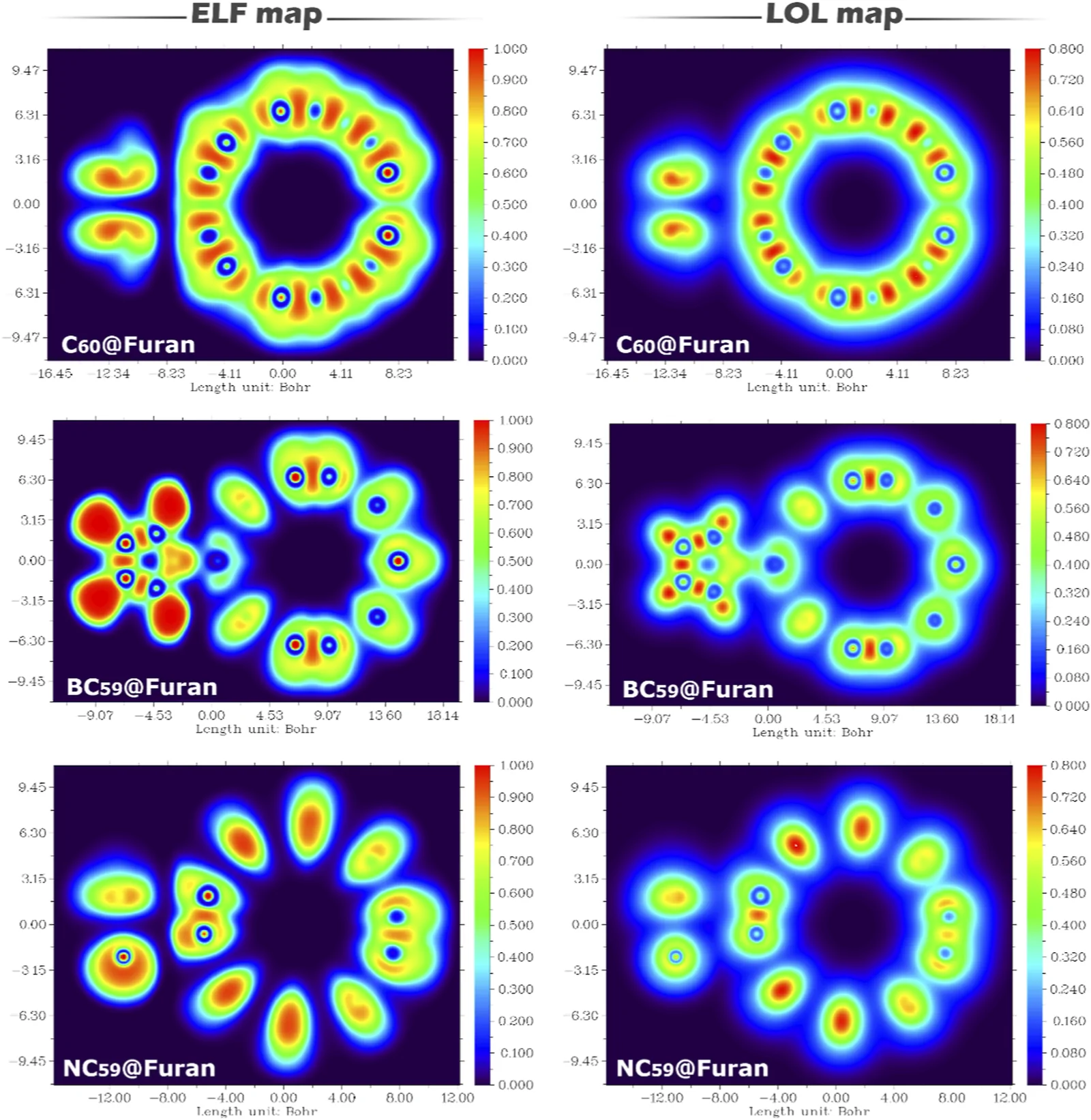
ELF and LOL maps presenting information about the electron localization within the C_60_@Furan, BC_59_@Furan and NC_59_@Furan complexes. The ELF and LOL Maps indicate that electrons are localized in red regions (close to 1), are moderately localized in yellow and green regions, and that they have either a low electron density (delocalized) in blue regions of the map.

For the C_60_@Furan system, both ELF and LOL maps show that electron localization is mainly concentrated on the fullerene framework, while the furan molecule remains relatively separated with limited overlap. The absence of significant red or yellow bridges between C_60_ and furan indicates weak interaction, consistent with physisorption. The interaction is dominated by van der Waals forces, with no clear evidence of strong orbital overlap or covalent character.

In the BC_59_@Furan system, the ELF and LOL contours show a pronounced increase in electron localization in the interaction region between the boron-doped site and furan. The emergence of more intense yellow-to-red regions between the adsorbent and the molecule indicates enhanced electron sharing. This suggests that boron doping creates an electron-deficient site that promotes stronger interaction with furan, resulting in partial charge transfer and more pronounced adsorption than with pristine C_60_.

In NC_59_@Furan, electron localization remains largely confined to the doped cage and the furan molecule, with only moderate changes near the interface. Nitrogen substitution alters the local electron distribution, but interfacial localization is less pronounced than in BC_59_@Furan. This behavior aligns with the smaller adsorption-induced electronic changes predicted for NC_59_@Furan.

### 3.8 Quantum theory of atoms in molecules analysis

Quantum Theory of Atoms in Molecules (QTAIM) analysis was used to characterize the intermolecular bond critical points (BCP) formed between furan and the pristine or doped fullerene cages (Figure 9). The principal topological descriptors considered were the electron density, ρ(r); the Laplacian of the electron density, ∇^2^ρ(r); the kinetic-energy density, G(r); the potential-energy density, V(r); and the total energy density, H(r) = G(r) + V(r). The electron density reflects the degree of electron accumulation at the bond critical point, whereas the signs and relative magnitudes of ∇^2^ρ(r), V(r), G(r), and H(r) provide complementary information about the nature of the interaction (Matta, 2013). The calculated parameters are presented in Table 6.

**FIGURE 9:**
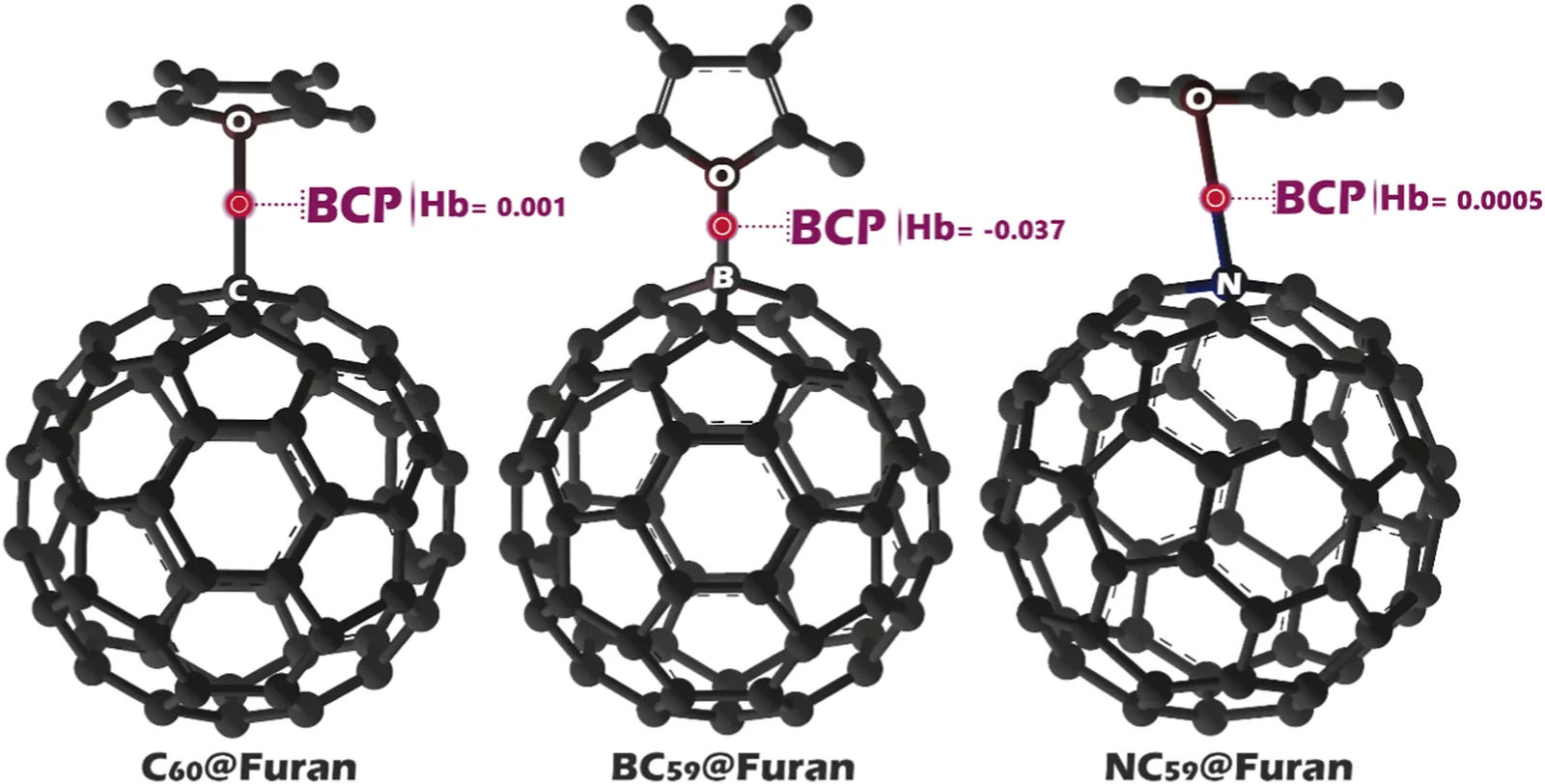
Calculated Hb values for each of the complexes designed in BCP.

For C_60_@Furan, both computational methods predict low electron-density values at the intermolecular BCP, with ρ(r) values of 0.007 and 0.006 a.u. at the B97D and ωB97X-D levels, respectively. The positive Laplacian values of 0.020 a.u., together with the small positive H(r) values of 0.001 a.u., indicate electron depletion at the BCP and a weak closed-shell interaction. The relatively small magnitudes of V(r) and G(r) are also consistent with a weak noncovalent contact dominated primarily by dispersion and electrostatic contributions.

A similar QTAIM pattern is obtained for NC_59_@Furan. The electron density at the intermolecular BCP is low, with ρ(r) values of 0.008 and 0.007 a.u. using B97D and ωB97X-D, respectively. The Laplacian remains positive, with values of 0.028 and 0.024 a.u., while H(r) is slightly positive at approximately 0.0005 a.u. These results identify the NC_59_···furan contact as a predominantly closed-shell, noncovalent interaction. Although the electron density is marginally higher than that obtained for C_60_@Furan at the B97D level, the small positive total energy density indicates that substantial covalent electron sharing is not predicted.

In contrast, BC_59_@Furan exhibits substantially greater electron accumulation at the intermolecular B···O bond critical point. The calculated ρ(r) values increase to 0.067 a.u. at the B97D level and 0.076 a.u. at the ωB97X-D level, approximately one order of magnitude larger than those obtained for C_60_@Furan and NC_59_@Furan. The potential-energy density is also considerably more negative, reaching −0.121 and −0.166 a.u., respectively, indicating a pronounced stabilizing contribution at the B···O interaction region.

## 4 Discussion

The discussion integrates experimental feasibility, possible synthesis routes, and comparisons with previous literature, while relating the structural, electronic, energetic, and topological findings to furan adsorption and the theoretically proposed single-use detection potential. These modifications may influence the calculated adsorption behavior and adsorption-induced electronic response toward furan. However, they should not be interpreted as evidence of experimental sensitivity or selectivity.

Comparison with the experimental IR spectrum reported by Mauron et al. shows very good agreement in terms of both the number and positions of the main absorption bands (Mauron et al., 2012). Experimentally, pristine C_60_ exhibits four characteristic IR-active modes located approximately at 1,428, 1,183, 577, and 528 cm^−1^. The calculated spectrum reproduces these features with only small frequency deviations, which are typical of harmonic DFT calculations and arise from neglect of anharmonicity and solid-state effects. Importantly, the relative intensity pattern and the absence of additional spurious bands confirm that the computational method correctly captures the symmetry and vibrational behavior of the C_60_ cage. This agreement provides a limited consistency check for the vibrational description of pristine C_60_. For the substituted structures, BC59 and NC59, the calculated IR spectra exhibit a clear increase in the number of active vibrational modes compared with pristine C_60_. This behavior is expected because substitution of one carbon atom by B or N removes the high Ih symmetry of the C_60_ cage, lifts the degeneracy of several cage vibrations, and activates modes that are IR-inactive or very weak in pristine C_60_. In both calculated spectra, additional bands are observed mainly in the 1,000–1,600 cm^−1^ region, together with shifts and intensity variations below approximately 600 cm^−1^. These changes are associated with perturbed C-C stretching, cage-deformation vibrations, and contributions from the newly introduced heteroatom-carbon bonds.

The calculated behavior of BC_59_ can be compared qualitatively with the experimental FTIR spectrum of B-doped C_60_ thin films reported by Zou et al. (2000). In that experimental study, pristine C_60_ exhibited its four principal bands at approximately 527, 576, 1,183, and 1,428 cm^−1^, whereas the B-doped films contained several additional absorptions, including bands near 545, 640, 731, 885, 1,007, 1,197, and 1,616 cm^−1^. The appearance of these additional experimental bands is qualitatively consistent with the greater number of vibrational features predicted for BC59 in the present calculations, particularly in the low-frequency cage-deformation region and the 1,000–1,600 cm^−1^ stretching region. Both the experimental and calculated results therefore suggest that boron incorporation lowers the cage symmetry and perturbs the original vibrational modes of C_60_. However, the experimental samples consisted of B-doped C_60_ films prepared by plasma-assisted deposition and also contained contributions from polymerized fullerene species. Consequently, an exact band-by-band correspondence with the present isolated substitutional BC_59_ model should not be expected.

For the nitrogen-containing system, the closest experimentally characterized compounds are azafullerene derivatives such as bisazafullerene, (C_59_N)_2_, and hydroazafullerene, C_59_HN, because neutral monomeric C_59_N generally undergoes stabilization or dimerization under bulk experimental conditions. Experimental IR analysis of (C_59_N)_2_ showed splitting of the principal C_60_-derived modes, activation of cage modes that are forbidden in highly symmetric C_60_, and characteristic absorptions at approximately 819, 836, and 844 cm^−1^ attributed to coupled C-N and C-C vibrations (Krause et al., 2001). Experimental C_59_HN spectra similarly showed splitting of the four strong C_60_ F_1_u-derived bands as a consequence of symmetry reduction (Tagmatarchis et al., 2006). The calculated NC_59_ spectrum in the present work exhibits the same principal qualitative response: retention of the fullerene cage vibrations accompanied by mode splitting, frequency shifts, intensity redistribution, and activation of additional bands following nitrogen substitution.

Nevertheless, the present NC_59_ model represents an isolated substitutional monomer treated using harmonic DFT calculations and a continuum solvent model, whereas the available experimental spectra correspond mainly to solid-state, dimeric, or hydrogen-stabilized azafullerenes. Differences in molecular form, intermolecular coupling, crystal packing, anharmonicity, temperature, and instrumental conditions can therefore produce differences in individual frequencies and relative intensities. Accordingly, the comparison should be interpreted as qualitative experimental support for the predicted symmetry-breaking and mode-activation effects of B and N substitution rather than as exact quantitative validation of the calculated BC_59_ and NC_59_ frequencies. A numerical comparison between the calculated IR main bands and the experimentally reported vibrational frequencies of the pristine and heteroatom-containing fullerene systems is presented in Supplementary Table S3 in the Supplementary Data.

The differences between pristine C_60_ and the doped cages are relatively small, amounting to approximately 1.2–1.4 kcal.mol^−1^ atom^−1^. Therefore, within the limitations of the present computational model, heteroatom substitution is predicted to cause only a minor weakening of the fullerene bonding network rather than substantial cage destabilization. The retained closed-cage geometries after optimization and the absence of imaginary vibrational frequencies provide complementary evidence that BC_59_ and NC_59_ correspond to local minima on the calculated potential-energy surface. Overall, the optimized closed-cage structures and absence of imaginary frequencies indicate that BC_59_ and NC_59_ are suitable local-minimum models for the subsequent theoretical analyses. Their practical stability and experimental accessibility nevertheless require empirical evaluation.

Experimental literature provides evidence that heteroatom incorporation into fullerene-based materials is feasible, although the experimentally obtained products do not always correspond to isolated neutral C_59_X monomers. Boron-containing C_60_ films have been prepared by radio-frequency plasma-assisted chemical vapor deposition and plasma-assisted vapor deposition. In these methods, energetic boron-containing species generated in the plasma interact with vaporized C_60_ and produce B-doped fullerene films. Spectroscopic measurements confirmed boron-induced modification of the C_60_ structure, although plasma-polymerized fullerene species may also be formed. Therefore, RF plasma-assisted deposition represents a plausible experimental route for preparing a BC_59_-like doped fullerene material, but additional mass-spectrometric, XPS, NMR, and structural characterization would be required to verify the formation and abundance of the specific substitutional BC_59_ cage considered in the present model (See reference 39).

Nitrogen-substituted fullerene chemistry is experimentally more established. The neutral C_59_N monomer is an open-shell species and tends to undergo stabilization through dimerization or chemical functionalization. C_59_N has been experimentally isolated in bulk as the azafullerene dimer (C_59_N)_2_, while hydrogen-stabilized C_59_HN has also been synthesized and characterized. Monomeric C_59_N units have additionally been stabilized through chemisorption on silicon surfaces. Therefore, practical realization of the NC_59_ model may require surface immobilization, ionic stabilization, hydrogen termination, functionalization, or use of the experimentally accessible azafullerene dimer rather than isolation of a free neutral NC_59_ monomer (Hummelen et al., 1995).

Consequently, BC_59_ and NC_59_ in the present study should be regarded as idealized substitutional molecular models representing the local electronic effects of B and N incorporation. The calculated results provide pre-synthesis guidance for selecting experimentally realizable B-doped fullerene films, stabilized azafullerene derivatives, or surface-supported heterofullerene structures for subsequent furan adsorption and electrochemical testing.

The B97D-calculated HOMO-LUMO gap of pristine C_60_ is 1.67 eV. This value is close to the previously reported literature value of 1.50 eV, corresponding to an absolute difference of 0.17 eV (Oshiyama et al., 1992). This comparison is included only as a limited consistency check for pristine C_60_ and should not be interpreted as experimental validation of the present computational model or of the calculated energy gaps for BC_59_, NC_59_, and their furan complexes. Moreover, because Kohn–Sham HOMO-LUMO gaps are functional-dependent and are not necessarily equivalent to experimentally measured optical or fundamental gaps, the present analysis focuses primarily on relative adsorption-induced changes and qualitative trends. Although B97D and ωB97XD produce different absolute energy-gap values, both functionals predict comparable qualitative trends across the studied systems (see Figure 3A).

Among the three systems, BC_59_@Furan exhibits the largest adsorption-induced reduction in Egap at the B97D level, together with a decrease in chemical hardness and an increase in chemical softness. These changes, combined with the short-calculated interaction distance and the complementary NBO and QTAIM results reported in the NBO and QTAIM analyses, suggest a pronounced electronic response upon furan adsorption. ECT is not used to rank the complexes or determine interaction strength; its negative sign is considered only as a qualitative indication that the predicted charge-transfer direction is from furan toward the fullerene-based adsorbent.

The predicted electronic-response changes do not account for experimental factors such as electrode-molecule contact resistance, surface defects, solvent dynamics, or environmental interferences, all of which could significantly affect real-world sensing performance. Therefore, while our calculations identify BC_59_ as a structurally promising candidate worthy of further investigation, we refrain from making definitive claims regarding its practical superiority. Instead, we propose that BC_59_ be considered a theoretically motivated lead for future experimental validation, particularly in the context of furan detection. We encourage systematic electrochemical measurements (e.g., cyclic voltammetry, impedance spectroscopy) and surface-characterization studies to verify the predicted sensitivity and selectivity under realistic operating conditions.

Overall, there is good qualitative agreement between the B97D and ωB97XD results. Both functionals predict the same trends in adsorption strength, recovery time, and conductivity changes, although ωB97XD generally yields stronger adsorption energies and longer recovery times due to its more accurate treatment of dispersion interactions. However, the recovery time analysis reveals that none of the studied systems exhibit the rapid desorption required for practical reversible sensing applications at room temperature. BC_59_ shows the strongest adsorption and largest conductivity enhancement but at the cost of essentially irreversible binding (τ ≈ 10^13^–10^24^ s). This makes BC_59_ a candidate for further experimental work as a permanent adsorbent for furan removal.

For electrochemical sensing evaluation, BC_59_ could be investigated as a theoretically proposed single-use detection platform in which the furan-induced electrical response is measured without requiring platform regeneration. This approach would be suitable for applications such as low-cost field-testing kits, environmental screening, or rapid preliminary assessments where reusability is not a primary requirement. However, experimental validation is essential to confirm whether the predicted conductivity changes are sufficient for practical detection limits and selectivity against interfering substances.

The most significant differentiation is observed in the π→π* transitions, which are crucial for charge mobility in conjugated systems. The E^2^ value for BC_59_@Furan (14.16 kcal.mol^−1^) is nearly twice as large as the values for C_60_@Furan (7.22 kcal.mol^−1^) and NC_59_@Furan (7.30 kcal.mol^−1^). This substantial increase demonstrates a much stronger coupling between the π-bonding and π-antibonding orbitals in the boron-doped system. The similar energy gaps [E(j)-E(i)] and Fock matrix elements [F (i,j)] across all complexes for this transition suggest that the dramatic increase in E^2^ for BC_59_ is not due to orbital energetics but rather to a fundamental enhancement in the electron-donating capability of the π-system induced by the electron-deficient boron atom.

This trend is further amplified in the lone pair (LP) to π* interactions. The stabilization energy for the LP→π* transition in BC_59_@Furan is exceptionally high at 61.81 kcal.mol^−1^, vastly overshadowing the values in C_60_@Furan (32.40 kcal.mol^−1^) and NC_59_@Furan (27.96 kcal.mol^−1^). This indicates an extremely efficient donation of electron density from lone pairs into the π-system of the fullerene cage in the presence of boron. The collective strength of these interactions (the significantly higher E^2^ values for σ→σ, π→π, and LP→π transitions) paints a coherent picture of a highly polarized and electronically responsive material. This electron-density redistribution within BC_59_ is consistent with its predicted electron-accepting role. In agreement with this qualitative interpretation, the negative sign of ECT indicates a predicted charge-transfer direction from furan to BC_59_; however, the numerical ECT magnitude is not interpreted as the amount or strength of charge transfer.

The negative ECT sign indicates a predicted charge-transfer direction from furan toward the BC_59_-based adsorbent according to the adopted convention. The NBO analysis provides the mechanistic underpinning for this: the strong π(C1-C4)→π*(C15-C16) interaction, among other high E^2^ transitions, reflects a significant redistribution of electron density within the BC_59_ framework upon furan adsorption. This internal polarization is qualitatively consistent with the predicted electron-accepting character of the BC_59_ model and with the charge-transfer direction inferred from the negative ECT sign. Therefore, the NBO and ECT results provide complementary qualitative indications of adsorption-induced polarization; however, neither analysis is interpreted as a direct quantitative measurement of transferred charge.

Taken together, the ELF and LOL maps are qualitatively consistent with the calculated interaction-strength order C_60_@Furan < NC_59_@Furan < BC_59_@Furan. However, these maps provide qualitative evidence of electron localization and should not be interpreted as direct quantitative measures of adsorption strength or charge transfer.

The positive ∇^2^ρ(r) values of 0.204 and 0.372 a.u. indicate that the BC_59_@Furan interaction does not exhibit the conventional charge-concentration pattern expected for a purely shared-shell covalent bond. However, unlike the C_60_@Furan and NC_59_@Furan complexes, BC_59_@Furan has negative total energy densities of −0.032 and −0.036 a.u. The combination of a positive Laplacian and negative H(r) is characteristic of an intermediate interaction with a significant stabilizing and partial covalent contribution. Accordingly, the short-calculated B···O contact should be described as a strongly polarized, partially covalent interaction rather than as either a conventional covalent bond or a hydrogen bond.

The ratio |V(r)|/G(r) further supports this interpretation. For BC_59_@Furan, the approximate ratios are 1.36 and 1.29 at the B97D and ωB97X-D levels, respectively. Values between one and 2 are generally associated with intermediate interactions containing both closed-shell and shared-shell characteristics. By comparison, the |V(r)|/G(r) ratios for C_60_@Furan and NC_59_@Furan are close to or below unity, supporting their predominantly closed-shell and noncovalent character.

Overall, the two functionals provide the same qualitative QTAIM classification. C_60_@Furan and NC_59_@Furan are characterized by low ρ(r), positive ∇^2^ρ(r), and slightly positive H(r), which are consistent with weak closed-shell interactions. BC_59_@Furan exhibits substantially higher electron density, a much more negative potential-energy density, and negative H(r), indicating a stronger, localized B···O interaction with partial covalent character. These results support the conclusion that boron substitution produces the most electronically significant interaction with furan among the investigated fullerene models.

As summarized in Table 7, the present BC_59_@Furan complex exhibits stronger calculated adsorption than the previously reported furan systems based on X_12_Y_12_ nanoclusters and metal-doped SWCNTs. At the B97D level, its adsorption energy of −34.61 kcal.mol^−1^ is more negative than the strongest values reported for Al_12_N_12_@Furan and Rh-doped SWCNT@Furan. The closest comparison is the BC_59_@Acetone system studied by Alamri and Alafnan under similar computational conditions. Although BC_59_@Furan shows a more negative adsorption energy than BC_59_@Acetone, acetone produces a shorter B···O distance and greater changes in the energy gap and dipole moment. These differences indicate that adsorption strength and electronic response depend strongly on the analyte structure and should not be considered equivalent measures of sensing performance. Overall, the comparison of sources suggests that BC_59_ may be a desirable adsorption platform for Furan; however, its extremely long predicted recovery time makes it more suitable for permanent adsorption or single-use detection than for rapid, reusable sensing applications. Because the cited studies employed different computational methods, structural models, and environmental conditions, these comparisons should be interpreted qualitatively. Experimental investigation is required to determine whether the predicted differences are retained under practical conditions.

**TABLE 7:** Comparison of the results of this work with previous theoretical studies on the adsorption performance of C_60_-based nanostructures.

Study	Analyte	Investigated material	Computational approach	Most relevant adsorption result	Principal electronic or interaction result	Proposed application
Shokuhi Rad (2016)	Furan	Al_12_N_12_, Al_12_P_12_, B_12_N_12_, and B_12_P_12_ nanoclusters	B3LYP and ωB97XD calculations	Eads ranged from -4.37 to -23.52 kcal.mol ^−^ ^1^. Al_12_N_12_ showed the strongest adsorption, with Eads = -23.52 kcal.mol ^−^ ^1^.	B_12_N_12_ produced the greatest adsorption-induced electronic-structure change and was identified as the most electronically responsive structure.	Al_12_N_12_ was proposed mainly as an adsorbent, whereas B_12_N_12_ was suggested as a candidate sensing material.
Yuksel et al. (2022)	Furan	Pd-, Ag-, and Rh-doped (8,0) SWCNTs	ωB97XD/6-31G(d,p)/LANL2DZ	Adsorption enthalpies were -11.85, -15.03, and -15.37 kcal.mol ^−^ ^1^ for Pd-, Ag-, and Rh-doped SWCNTs, respectively.	Furan adsorption produced slight decreases in the HOMO-LUMO gaps, moderate conductivity increases, and electron transfer from furan toward the metal-doped nanotubes.	The metal-doped SWCNTs were suggested as potential adsorbents and candidate sensing platforms for furan.
Alamri and Alafnan, (2025)	Acetone	C_60_, BC_59_, NC_59_, and SiC_59_ nanocages	B97D/6-31G(d), CPCM water model, and BSSE correction	BC_59_@Acetone: Eads = -26.12 kcal.mol^-1^. SiC_59_@Acetone showed the strongest adsorption, with Eads = -32.78 kcal.mol ^−^ ^1^.	BC_59_ formed a short B···O contact of approximately 1.60 Å. SiC_59_ showed the strongest combined adsorption and electronic response, including a final energy gap of 0.74 eV and a dipole moment of 19.55 D.	BC_59_ was mainly proposed for acetone adsorption/removal, whereas SiC_59_ was identified as the most promising theoretically proposed reusable sensing platform.
Present work, B97D	Furan	C_60_, BC_59_, NC_59_, and SiC_59_ nanocages	B97D/6-31G(d), CPCM water model	Eads values were -24.36, -34.61, and -28.54 kcal.mol ^−^ ^1^ for C60@Furan, BC59@Furan, and NC59@Furan, respectively.	BC_59_@Furan showed the shortest interaction distance of approximately 1.75 Å, an energy-gap reduction from 1.35 to 1.09 eV, an increase in dipole moment from 1.50 to 8.69 D, and the most pronounced electronic perturbation among the present systems.	Because of its extremely long predicted recovery time, BC_59_ is more appropriately considered a permanent adsorbent or a theoretically proposed disposable/single-use sensing platform rather than a reusable system.
Present work, ωB97XD	Furan	C_60_, BC_59_, and NC_59_ nanocages	ωB97XD/6-31G(d), CPCM water model	Eads values were -21.86, -49.44, and -27.08 kcal.mol ^−^ ^1^ for C_60_@Furan, BC_59_@Furan, and NC_59_@Furan, respectively.	The ωB97XD results preserved the B97D adsorption-strength order and predicted particularly strong and effectively irreversible furan binding on BC_59_.	The results support BC_59_ as a candidate permanent adsorbent or single-use detection material requiring experimental validation.

## 5 Conclusion

This study theoretically evaluated pristine C_60_ and its B- and N-substituted derivatives, BC_59_ and NC_59_, as candidate materials for furan adsorption and adsorption-induced electronic response. Calculations were performed using the dispersion-corrected B97D and ωB97X-D functionals with the 6–31G(d) basis set. Both computational approaches predicted that heteroatom substitution modifies the geometry, electron distribution, and interaction of the fullerene cage with furan.

Among the investigated complexes, BC_59_@Furan exhibited the strongest calculated adsorption, with Eads values of −34.61 and −49.44 kcal.mol^−1^ at the B97D and ωB97X-D levels, respectively. It also showed the shortest intermolecular contact, approximately 1.75 Å, and the largest adsorption-induced reduction in the HOMO-LUMO gap. At the B97D level, the gap decreased from 1.35 to 1.09 eV, while the chemical softness increased from 0.74 to 0.91 eV^-1^. The negative ECT sign predicted electron transfer from furan toward BC_59_ according to the adopted convention; however, the ECT magnitude was not interpreted as the amount of transferred charge. NBO, NCI/RDG, ELF/LOL, and QTAIM analyses consistently indicated a stronger and more localized calculated interaction in BC_59_@Furan than in C_60_@Furan and NC_59_@Furan.

The predicted desorption times reveal an important limitation. BC_59_@Furan exhibited exceptionally long τ values of approximately 4.46 × 10^13^ s and 1.26 × 10^24^ s at the B97D and ωB97X-D levels, respectively. C_60_@Furan and NC_59_@Furan also showed desorption times that are too long for rapid, repeatedly regenerable room-temperature sensing. Therefore, none of the investigated structures can presently be recommended as a reusable sensing platform. BC_59_ may instead be considered a candidate permanent adsorbent or a theoretically proposed single-use detection platform, provided that a measurable and selective experimental response can be demonstrated.

The cohesive-energy and frequency analyses indicate that the optimized BC_59_ and NC_59_ models correspond to closed-cage local minima, although these calculations do not establish their thermodynamic synthesizability or long-term experimental stability. The isolated substituted cages should therefore be regarded as idealized pre-synthesis models representing the local electronic effects of B and N incorporation. Therefore, experimental evaluation may initially focus on RF-plasma-prepared B-doped C_60_ films and on stabilized azafullerene forms, such as surface-supported C_59_N, C_59_HN, or the experimentally isolated (C_59_N)_2_ dimer. The isolated neutral BC_59_ and NC_59_ cages used here should therefore be understood as idealized pre-synthesis models whose practical form and stability must be established experimentally.

Overall, the calculations identify BC_59_ as the most promising theoretical candidate among the investigated systems for further furan adsorption and single-use detection studies. Nevertheless, the results arise from idealized molecular models and do not include explicit solvent molecules, finite-temperature dynamics, electrode-molecule contacts, surface defects, competitive adsorption, or real-matrix interference. Experimental synthesis, structural characterization, adsorption measurements, selectivity tests, and electrochemical evaluation are required before any practical application can be established.

## Data Availability

The original contributions presented in the study are included in the article/Supplementary Material, further inquiries can be directed to the corresponding author.
