# Supplementary material for: In silico investigation of BC59 nanocage for furan adsorption and single-use detection potential
Source: Front Chem. 2026 Sep 30;14:1913300. doi: 10.3389/fchem.2026.1913300 (PMC13661588; doi:10.3389/fchem.2026.1913300)
Supplement: Supplementary file 1 [file Table1.docx]

In order to ensure the reproducibility of the results reported in this work, the XYZ coordinates for each of the designed structures were reported in Table S1.

Table S1. XYZ coordinates of the structures designed in this work

C_60_

C	-1.33865095	-0.89248321	0.00000000

C	0.12523805	-0.89248321	0.00000000

C	0.81752705	0.30671479	0.00000000

C	-2.03102895	0.30664579	0.00002100

C	-1.79103095	-1.99886821	-0.84514300

C	-0.60674495	-2.68262021	-1.36751300

C	0.57761505	-1.99882821	-0.84518700

C	1.69778605	-1.84612721	-1.64467700

C	2.00178205	0.46813779	-0.84525000

C	0.81743105	2.51939679	-0.84526500

C	0.12502805	3.41317479	-1.64470200

C	-1.33886495	3.41315179	-1.64464800

C	-2.03113095	2.51934779	-0.84515400

C	-3.21545295	1.83554079	-1.36745400

C	-3.21539095	0.46802379	-0.84510200

C	-3.64329695	-0.57847621	-1.64450700

C	-2.91129995	-1.84623521	-1.64452900

C	-0.60679695	-3.17672521	-2.66101900

C	-1.79112295	-3.01535721	-3.50614400

C	-2.91133695	-2.36859921	-3.01203600

C	-3.64335095	-1.42369121	-3.85720300

C	-4.09572095	-0.31733921	-3.01201000

C	-4.09577295	0.97617879	-3.50610800

C	-3.64340695	2.08255379	-2.66095700

C	-2.91152195	3.02751679	-3.50619100

C	-1.79129495	3.67431979	-3.01217300

C	2.00172105	1.83567179	-1.36763700

C	0.57728505	2.65785679	-5.67325800

C	1.69755505	2.50523479	-4.87388600

C	2.42952805	1.23748379	-4.87388000

C	2.00162605	0.19098879	-5.67330600

C	0.81729605	0.35236779	-6.51843700

C	-1.33898695	1.55147279	-6.51837200

C	-1.79135595	2.65783179	-5.67318700

C	-0.60704695	3.34163279	-5.15088600

C	-0.60700995	3.83573079	-3.85737800

C	0.57734905	3.67433579	-3.01221300

C	1.69757505	3.02759879	-3.50632700

C	2.88192305	0.97633379	-3.50637300

C	2.88198005	-0.31715621	-3.01226300

C	2.42958705	-1.42355721	-3.85740200

C	2.00165905	-1.17653621	-5.15092200

C	0.81737505	-1.86033221	-5.67323000

C	0.08537305	-0.91543221	-6.51838300

C	-1.29929695	-0.91544821	-6.51832700

C	-2.03128395	0.35230179	-6.51833200

C	-2.91155695	2.50513879	-4.87370400

C	-3.64345595	1.23734579	-4.87365800

C	-3.21557095	0.19086979	-5.67310300

C	-3.21551595	-1.17665821	-5.15073900

C	-2.03118895	-1.86040121	-5.67312600

C	-1.33880095	-2.75418021	-4.87371500

C	0.12509205	-2.75415421	-4.87374700

C	0.57750705	-3.01528621	-3.50626500

C	1.69772805	-2.36848721	-3.01222400

C	2.42955005	2.08271579	-2.66118400

C	2.42966505	-0.57832621	-1.64469900

C	0.12492105	1.55151079	-6.51840400

C	0.08554505	1.57447279	-0.00001900

C	-1.29912295	1.57444079	0.00000500

C60@Furan

C	2.34258065	2.31379039	-0.37173849

C	2.12245065	2.29601439	1.09943051

C	2.44761265	1.15868239	1.88419851

C	2.94887765	1.24030339	-1.02978449

C	1.22620065	3.02266839	-0.96444649

C	0.33314465	3.46161339	0.09296051

C	0.88620565	3.00779639	1.36304051

C	0.00900865	2.59601339	2.38906751

C	1.52138465	0.72407339	2.93187651

C	2.44763565	-1.16053261	1.88302651

C	2.12249465	-2.29707761	1.09711051

C	2.34262765	-2.31336461	-0.37407549

C	2.94890165	-1.23919961	-1.03103849

C	2.33139465	-0.73793261	-2.24570649

C	2.33138065	0.74025339	-2.24495949

C	1.25737565	1.42866939	-2.85000849

C	0.70331965	2.60238839	-2.20078949

C	-1.05368935	3.48278439	-0.11525849

C	-1.59920435	3.03929839	-1.39213249

C	-0.73509435	2.60634439	-2.41373749

C	-1.07482435	1.43019639	-3.20187849

C	0.15705065	0.70330039	-3.46739749

C	0.15706365	-0.69978961	-3.46810549

C	1.25740265	-1.42575861	-2.85144849

C	0.70336965	-2.60014461	-2.20341649

C	1.22625765	-3.02166261	-0.96749949

C	1.52139865	-0.72699961	2.93114351

C	-1.95244135	-3.04016261	0.93705851

C	-1.42555735	-2.60536261	2.16749451

C	-1.98894635	-1.43198161	2.82475251

C	-3.04534035	-0.72977961	2.22258651

C	-3.58482935	-1.17796061	0.94608751

C	-2.82801035	-2.31241861	-1.12567949

C	-1.59914635	-3.03792061	-1.39519949

C	-1.05362235	-3.48268461	-0.11877549

C	0.33321165	-3.46169461	0.08946451

C	0.88626165	-3.00914661	1.36000151

C	0.00905765	-2.59841661	2.38644351

C	-0.89784335	-0.70413461	3.45578451

C	-0.89785635	0.70062939	3.45649351

C	-1.98896935	1.42909239	2.82619351

C	-3.04535035	0.72747739	2.22331851

C	-3.58485635	1.17694339	0.94727651

C	-3.91563235	-0.00011461	0.15921051

C	-3.70979835	0.00058939	-1.23319449

C	-3.15742335	-1.17601861	-1.88675549

C	-0.73504435	-2.60391861	-2.41636749

C	-1.07479535	-1.42697961	-3.20331849

C	-2.26535035	-0.72667861	-2.94408249

C	-2.26536435	0.72960939	-2.94334849

C	-3.15744735	1.17786639	-1.88556949

C	-2.82805635	2.31350439	-1.12334649

C	-3.04635235	2.31290239	0.31908051

C	-1.95249835	3.03917739	0.94012751

C	-1.42560535	2.60314739	2.17012251

C	0.33568765	-1.42754061	3.18577151

C	0.33566065	1.42433439	3.18721151

C	-3.04630435	-2.31327461	0.31674551

C	3.07549265	-0.00062561	1.30324451

C	5.57611635	-1.13231739	-0.07952651

C	6.83967935	-0.72290839	0.21119349

C	6.83977735	0.72262661	0.21168749

C	5.57626135	1.13240461	-0.07872451

O	4.77993635	0.00016861	-0.27914451

H	5.05746035	-2.07488839	-0.19719951

H	7.68865635	-1.37351039	0.39899249

H	7.68884035	1.37298861	0.39992649

H	5.05773135	2.07512961	-0.19572751

C	3.53515865	0.00014239	-0.20816049

BC59

C	1.11656800	2.49133300	-2.32124100

C	-0.35603800	2.69426100	-2.29628900

C	-1.02293400	3.23905200	-1.16606900

C	1.92437600	2.88052600	-1.24457400

C	1.36836200	1.25364800	-3.02972300

C	0.09821000	0.69631900	-3.46798700

C	-0.96170300	1.58679200	-3.01179400

C	-2.19343500	1.04146200	-2.59597500

C	-2.28860600	2.65275100	-0.72707400

C	-1.02294100	3.23905400	1.16606000

C	-0.35605100	2.69426300	2.29628500

C	1.11655500	2.49133600	2.32124500

C	1.92436900	2.88052700	1.24458200

C	2.92464900	1.94830900	0.74211400

C	2.92465400	1.94830900	-0.74210000

C	3.18787500	0.74632500	-1.42759300

C	2.40181100	0.40025900	-2.60127700

C	-0.09663200	-0.69100200	-3.48519800

C	0.97148100	-1.57664000	-3.03940900

C	2.19614700	-1.03874700	-2.60397800

C	2.85503500	-1.58933300	-1.42787800

C	3.46220300	-0.48558100	-0.70174000

C	3.46219900	-0.48558000	0.70176000

C	3.18786700	0.74632700	1.42761100

C	2.40179600	0.40026100	2.60129100

C	1.36834500	1.25365100	3.02972900

C	-2.28861000	2.65275100	0.72705800

C	-1.36488300	-1.25107400	3.03834700

C	-2.39424000	-0.39643500	2.60350200

C	-3.18477700	-0.74803400	1.43057100

C	-2.90804500	-1.93179300	0.72883500

C	-1.83867800	-2.81231600	1.17751600

C	0.36360500	-2.67641200	2.31243400

C	0.97146300	-1.57663700	3.03941600

C	-0.09665200	-0.69099800	3.48519800

C	0.09819000	0.69632300	3.46798700

C	-0.96172100	1.58679500	3.01178700

C	-2.19345100	1.04146500	2.59596100

C	-3.47619700	0.47866300	0.70234000

C	-3.47619300	0.47866300	-0.70236100

C	-3.18476900	-0.74803500	-1.43058900

C	-2.90804100	-1.93179400	-0.72885000

C	-1.83867100	-2.81231700	-1.17752400

C	-1.17763000	-3.35212700	-0.00000200

C	0.21682600	-3.55088000	0.00000200

C	1.00059900	-3.21022000	1.17679600

C	2.19613200	-1.03874500	2.60399200

C	2.85502700	-1.58933200	1.42789600

C	2.26935000	-2.65796000	0.72804000

C	2.26935500	-2.65796000	-0.72802400

C	1.00060600	-3.21022100	-1.17678700

C	0.36361800	-2.67641400	-2.31243000

C	-1.08195300	-2.47623900	-2.31260900

C	-1.36486500	-1.25107700	-3.03835300

C	-2.39422500	-0.39643800	-2.60351600

C	-2.86717700	1.58427000	1.42515300

C	-2.86716900	1.58426900	-1.42517100

C	-1.08196600	-2.47623700	2.31260600

C	-0.28398400	3.66752300	-0.00000300

B	1.24195600	3.51730900	0.00000200

BC59@Furan

C	2.07519200	2.31645300	-0.38381300

C	1.86834100	2.29745700	1.08965500

C	2.20184100	1.16133800	1.87342800

C	2.67461400	1.24242200	-1.04953700

C	0.95375200	3.02552600	-0.96654400

C	0.07023900	3.46414300	0.09984800

C	0.63496900	3.01008200	1.36475000

C	-0.23134200	2.59708900	2.39891700

C	1.28739500	0.72594500	2.93019800

C	2.20203300	-1.16099300	1.87337300

C	1.86869500	-2.29711200	1.08952900

C	2.07554900	-2.31601500	-0.38394600

C	2.67479500	-1.24184900	-1.04960300

C	2.04812100	-0.73965500	-2.26091800

C	2.04801000	0.74017300	-2.26088300

C	0.96721400	1.42745000	-2.85236600

C	0.41907200	2.60149400	-2.19662200

C	-1.31814700	3.48329300	-0.09512400

C	-1.87552900	3.03858400	-1.36636900

C	-1.02139700	2.60498800	-2.39595200

C	-1.36855400	1.42866800	-3.18064700

C	-0.13954900	0.70170400	-3.45808900

C	-0.13944900	-0.70146100	-3.45812400

C	0.96743600	-1.42707600	-2.85244600

C	0.41947300	-2.60125600	-2.19678100

C	0.95422200	-3.02524600	-0.96671200

C	1.28750900	-0.72580000	2.93015700

C	-2.20610000	-3.03941800	0.96894100

C	-1.66797900	-2.60441800	2.19417700

C	-2.22480500	-1.43075500	2.85639900

C	-3.28687200	-0.72895300	2.26409000

C	-3.83864100	-1.17790600	0.99316200

C	-3.10123400	-2.31294300	-1.08548900

C	-1.87506000	-3.03869100	-1.36653200

C	-1.31760300	-3.48339200	-0.09531000

C	0.07078300	-3.46403600	0.09966200

C	0.63544300	-3.00997100	1.36459000

C	-0.23094500	-2.59713800	2.39875700

C	-1.12728400	-0.70248100	3.47597400

C	-1.12739100	0.70223800	3.47600900

C	-2.22504100	1.43038200	2.85648800

C	-3.28698900	0.72844000	2.26412900

C	-3.83879100	1.17735000	0.99321900

C	-4.17647800	-0.00027800	0.20887900

C	-3.98366500	-0.00023100	-1.18557300

C	-3.43776200	-1.17696300	-1.84404500

C	-1.02100600	-2.60493600	-2.39610900

C	-1.36834700	-1.42862000	-3.18074100

C	-2.55634800	-0.72818900	-2.91056100

C	-2.55645300	0.72804500	-2.91051500

C	-3.43793200	1.17662200	-1.84397900

C	-3.10158400	2.31261600	-1.08536100

C	-3.30653200	2.31289100	0.35912600

C	-2.20657400	3.03914700	0.96910900

C	-1.66838800	2.60418300	2.19434000

C	0.10362500	-1.42589100	3.19482100

C	0.10340700	1.42584200	3.19491000

C	-3.30618300	-2.31332100	0.35899300

C	2.82267200	0.00023800	1.28628300

C	5.91167700	-1.12236700	-0.11621800

C	7.16470500	-0.72211000	0.23911000

C	7.16506200	0.72168700	0.23893700

C	5.91223800	1.12248500	-0.11649900

O	5.13161400	0.00022500	-0.35344700

H	5.39708900	-2.06513500	-0.25390600

H	8.00092700	-1.37528800	0.47163400

H	8.00161000	1.37450600	0.47129300

H	5.39814000	2.06548700	-0.25440600

B	3.21530600	0.00031800	-0.22766700

NC59

C	1.11655600	2.49133800	-2.32124100

C	-0.35605100	2.69425900	-2.29628900

C	-1.02294900	3.23904700	-1.16606900

C	1.92436300	2.88053500	-1.24457400

C	1.36835600	1.25365400	-3.02972300

C	0.09820700	0.69631900	-3.46798700

C	-0.96171000	1.58678700	-3.01179400

C	-2.19344000	1.04145200	-2.59597500

C	-2.28861800	2.65274000	-0.72707400

C	-1.02295600	3.23904900	1.16606000

C	-0.35606400	2.69426100	2.29628500

C	1.11654300	2.49134100	2.32124500

C	1.92435600	2.88053600	1.24458200

C	2.92464000	1.94832300	0.74211400

C	2.92464500	1.94832300	-0.74210000

C	3.18787200	0.74634000	-1.42759300

C	2.40180900	0.40027000	-2.60127700

C	-0.09662900	-0.69100200	-3.48519800

C	0.97148800	-1.57663500	-3.03940900

C	2.19615200	-1.03873700	-2.60397800

C	2.85504200	-1.58932000	-1.42787800

C	3.46220500	-0.48556500	-0.70174000

C	3.46220100	-0.48556400	0.70176000

C	3.18786400	0.74634200	1.42761100

C	2.40179400	0.40027200	2.60129100

C	1.36833900	1.25365700	3.02972900

C	-2.28862200	2.65274000	0.72705800

C	-1.36487700	-1.25108000	3.03834700

C	-2.39423800	-0.39644600	2.60350200

C	-3.18477300	-0.74804900	1.43057100

C	-2.90803600	-1.93180700	0.72883500

C	-1.83866500	-2.81232500	1.17751600

C	0.36361800	-2.67641000	2.31243400

C	0.97147000	-1.57663200	3.03941600

C	-0.09664900	-0.69099800	3.48519800

C	0.09818700	0.69632300	3.46798700

C	-0.96172800	1.58679000	3.01178700

C	-2.19345600	1.04145500	2.59596100

C	-3.47619900	0.47864700	0.70234000

C	-3.47619500	0.47864700	-0.70236100

C	-3.18476500	-0.74805000	-1.43058900

C	-2.90803200	-1.93180800	-0.72885000

C	-1.83865800	-2.81232600	-1.17752400

C	-1.17761400	-3.35213300	-0.00000200

C	0.21684300	-3.55087900	0.00000200

C	1.00061400	-3.21021500	1.17679600

C	2.19613700	-1.03873500	2.60399200

C	2.85503400	-1.58931900	1.42789600

C	2.26936200	-2.65794900	0.72804000

C	2.26936700	-2.65794900	-0.72802400

C	1.00062100	-3.21021600	-1.17678700

C	0.36363100	-2.67641200	-2.31243000

C	-1.08194100	-2.47624400	-2.31260900

C	-1.36485900	-1.25108300	-3.03835300

C	-2.39422300	-0.39644900	-2.60351600

C	-2.86718400	1.58425700	1.42515300

C	-2.86717600	1.58425600	-1.42517100

C	-1.08195400	-2.47624200	2.31260600

C	-0.28400100	3.66752200	-0.00000300

N	1.24194000	3.51731500	0.00000200

NC59@Furan

C	2.07519200	2.31645300	-0.38381300

C	1.86834100	2.29745700	1.08965500

C	2.20184100	1.16133800	1.87342800

C	2.67461400	1.24242200	-1.04953700

C	0.95375200	3.02552600	-0.96654400

C	0.07023900	3.46414300	0.09984800

C	0.63496900	3.01008200	1.36475000

C	-0.23134200	2.59708900	2.39891700

C	1.28739500	0.72594500	2.93019800

C	2.20203300	-1.16099300	1.87337300

C	1.86869500	-2.29711200	1.08952900

C	2.07554900	-2.31601500	-0.38394600

C	2.67479500	-1.24184900	-1.04960300

C	2.04812100	-0.73965500	-2.26091800

C	2.04801000	0.74017300	-2.26088300

C	0.96721400	1.42745000	-2.85236600

C	0.41907200	2.60149400	-2.19662200

C	-1.31814700	3.48329300	-0.09512400

C	-1.87552900	3.03858400	-1.36636900

C	-1.02139700	2.60498800	-2.39595200

C	-1.36855400	1.42866800	-3.18064700

C	-0.13954900	0.70170400	-3.45808900

C	-0.13944900	-0.70146100	-3.45812400

C	0.96743600	-1.42707600	-2.85244600

C	0.41947300	-2.60125600	-2.19678100

C	0.95422200	-3.02524600	-0.96671200

C	1.28750900	-0.72580000	2.93015700

C	-2.20610000	-3.03941800	0.96894100

C	-1.66797900	-2.60441800	2.19417700

C	-2.22480500	-1.43075500	2.85639900

C	-3.28687200	-0.72895300	2.26409000

C	-3.83864100	-1.17790600	0.99316200

C	-3.10123400	-2.31294300	-1.08548900

C	-1.87506000	-3.03869100	-1.36653200

C	-1.31760300	-3.48339200	-0.09531000

C	0.07078300	-3.46403600	0.09966200

C	0.63544300	-3.00997100	1.36459000

C	-0.23094500	-2.59713800	2.39875700

C	-1.12728400	-0.70248100	3.47597400

C	-1.12739100	0.70223800	3.47600900

C	-2.22504100	1.43038200	2.85648800

C	-3.28698900	0.72844000	2.26412900

C	-3.83879100	1.17735000	0.99321900

C	-4.17647800	-0.00027800	0.20887900

C	-3.98366500	-0.00023100	-1.18557300

C	-3.43776200	-1.17696300	-1.84404500

C	-1.02100600	-2.60493600	-2.39610900

C	-1.36834700	-1.42862000	-3.18074100

C	-2.55634800	-0.72818900	-2.91056100

C	-2.55645300	0.72804500	-2.91051500

C	-3.43793200	1.17662200	-1.84397900

C	-3.10158400	2.31261600	-1.08536100

C	-3.30653200	2.31289100	0.35912600

C	-2.20657400	3.03914700	0.96910900

C	-1.66838800	2.60418300	2.19434000

C	0.10362500	-1.42589100	3.19482100

C	0.10340700	1.42584200	3.19491000

C	-3.30618300	-2.31332100	0.35899300

C	2.82267200	0.00023800	1.28628300

C	5.91167700	-1.12236700	-0.11621800

C	7.16470500	-0.72211000	0.23911000

C	7.16506200	0.72168700	0.23893700

C	5.91223800	1.12248500	-0.11649900

O	5.13161400	0.00022500	-0.35344700

H	5.39708900	-2.06513500	-0.25390600

H	8.00092700	-1.37528800	0.47163400

H	8.00161000	1.37450600	0.47129300

H	5.39814000	2.06548700	-0.25440600

N	3.21530600	0.00031800	-0.22766700

Figure 1S illustrates the evolution of the total electronic energy during geometry optimization of C_60_, BC_59_, NC_59_, and furan. All structures exhibit a pronounced energy reduction during the initial optimization steps, followed by a nearly horizontal region, indicating progressive structural relaxation and numerical convergence. C_60_ undergoes its main energy decrease in the first optimization step and reaches an approximately constant value after the second step. Furan shows a similarly rapid relaxation, with only negligible energy changes after approximately three steps. For NC_59_, the energy decreases over several successive steps before stabilizing at around the fourth step, suggesting that nitrogen substitution induces additional geometrical rearrangement relative to pristine C_60_. BC_59_ exhibits the most gradual convergence profile and requires more optimization cycles; however, its energy becomes essentially constant after approximately four steps, with the remaining iterations likely associated with satisfying the gradient and displacement convergence criteria. Overall, the terminal plateaus and absence of persistent energy oscillations support the successful convergence of the geometry optimizations.

Fig S1. Evolution of the total electronic energy during the geometry optimization of C_60_, BC_59_, NC_59_, and furan.

Statistical validation is an essential step in computational chemistry because it evaluates the reliability, reproducibility, and consistency of results obtained from different theoretical methods. In the present study, statistical analyses were performed to compare the adsorption-related properties predicted by the B97D and ωB97XD functionals, including adsorption energy, dipole moment, energy gap, recovery time, and electrical conductivity. Since density functional theory (DFT) calculations are inherently functional-dependent, statistical comparison helps determine whether the observed trends are method-independent and therefore physically meaningful. Strong statistical agreement between two independent computational approaches increases confidence in the robustness of the predicted sensing behavior and supports the validity of the reported conclusions.

To quantitatively evaluate the consistency between the two functionals, Pearson correlation coefficients (r) and Spearman rank correlation coefficients (ρ) were calculated (Equations 1 and 2). The Pearson correlation coefficient (r) measures the degree of linear relationship between two datasets and ranges from -1 to +1. Values close to +1 indicate a strong positive linear correlation, meaning that both computational methods produce highly similar numerical trends. In contrast, the Spearman rank correlation coefficient (ρ) evaluates the monotonic relationship between datasets based on ranking rather than absolute numerical values. A Spearman coefficient of +1 indicates identical ranking behavior between the two methods, demonstrating that both functionals predict the same qualitative ordering of sensor performance even if their absolute values differ. Therefore, the combined use of Pearson r and Spearman ρ provides a comprehensive statistical framework for assessing both quantitative agreement and qualitative consistency between the B97D and ωB97XD methods.

The Pearson correlation coefficient (r) measures the strength of the linear relationship between two datasets and is expressed as (Equation 1):

$$\boldsymbol{r=}\frac{\sum_{\boldsymbol{i=1}}^{\boldsymbol{n}} \left( \boldsymbol{x}_{\boldsymbol{i}}\boldsymbol{-}\bar{\boldsymbol{x}} \right)\left( \boldsymbol{y}_{\boldsymbol{i}}\boldsymbol{-}\bar{\boldsymbol{y}} \right)}{\sqrt{\sum_{\boldsymbol{i=1}}^{\boldsymbol{n}} \left( \boldsymbol{x}_{\boldsymbol{i}}\boldsymbol{-}\bar{\boldsymbol{x}} \right)^{\boldsymbol{2}}}\sqrt{\sum_{\boldsymbol{i=1}}^{\boldsymbol{n}} \left( \boldsymbol{y}_{\boldsymbol{i}}\boldsymbol{-}\bar{\boldsymbol{y}} \right)^{\boldsymbol{2}}}}$$

(1)

where r is the dimensionless Pearson correlation coefficient; x_i_ and y_i_ are the ith paired values obtained using the B97D and ωB97XD functionals, respectively; $\bar{x}$ and $\bar{y}$ are the arithmetic means of the B97D and ωB97XD datasets, respectively; i is the observation index; n is the total number of paired observations; and $\sqrt{\sum\begin{matrix} n \\ i=1 \end{matrix}}$ denotes summation over all paired observations. The value of r ranges from -1 to +1.

The Spearman rank correlation coefficient (ρ) evaluates the similarity in ranking/order between two datasets and is given by (Equation 2):

$$\boldsymbol{\rho=1-}\frac{\boldsymbol{6}\sum\boldsymbol{d}_{\boldsymbol{i}}^{\boldsymbol{2}}}{\boldsymbol{n(}\boldsymbol{n}^{\boldsymbol{2}}\boldsymbol{-1)}}$$

(2)

where ρ is the dimensionless Spearman rank correlation coefficient; $d_{i}$ is the difference between the ranks assigned to the ith paired B97D and ωB97XD values; $d_{i}^{2}$ is the squared rank difference; i is the observation index; n is the number of paired observations; and $\sum d_{i}^{2}$ is the sum of the squared rank differences over all observations. The value of ρ ranges from -1 to +1, with ρ=+1 indicating identical ranking by the two functionals.

| Table S2. Pearson (r) and Spearman (ρ) correlation coefficients for adsorption energy, dipole moment, energy gap, recovery time, and conductivity-related quantity calculated using the B97D and ωB97XD functionals. | | |
| --- | --- | --- |
| Property | **Pearson r** | **Spearman ρ** |
| Adsorption energy (\|Eads\|) | 0.972 | 1.000 |
| Dipole moment | 0.986 | 1.000 |
| Energy gap (Egap) | 0.998 | 1.000 |
| Recovery time [Log_10_(τ)] | 0.972 | 1.000 |
| Electrical conductivity (σ) | 0.998 | 1.000 |

The statistical results show a very good agreement between the B97D and ωB97XD functionals for all the adsorption parameters considered. In particular, the Spearman rank correlation coefficient (ρ = 1.000) obtained for each parameter indicates that both methods predict exactly the same qualitative ranking of the performance of the designed structures (Table S2). Similarly, the very high Pearson correlation coefficients (r = 0.972-0.998) confirm the strong quantitative agreement between the two computational approaches (Fig S1-(a-e)). These findings indicate that although the two functionals may produce different absolute numerical values, especially for properties such as the HOMO-LUMO gap and conductivity, they consistently reproduce the same physical trends and adsorption behavior. Overall, the statistical analysis supports the robustness of the qualitative conclusion that BC_59_ exhibits the strongest calculated adsorption and the largest adsorption-induced electronic response among the investigated fullerene systems (Fig S2).

Fig S2. Correlation plots comparing the adsorption-related parameters calculated using the B97D and ωB97XD functionals for (a) adsorption energy (Eads), (b) dipole moment (μ), (c) energy gap (Egap), (d) recovery time (log_10_(τ)), and (e) electrical conductivity (σ). The strong linear correlations and identical rank-order behavior demonstrate excellent consistency between the two computational methods. The dashed red lines represent the linear regression fits.

Table S2 provides a numerical comparison between the principal calculated IR bands and experimentally reported vibrational frequencies of pristine and heteroatom-containing fullerene systems. For pristine C_60_, the calculated bands at approximately 1451, 1190, 575, and 523 cm^-1^ correspond closely to the experimental bands at 1428, 1183, 576, and 527 cm^-1^, respectively. The absolute deviations range from approximately 1 to 23 cm^-1^, indicating that the employed computational method reasonably reproduces the characteristic vibrational pattern of the C_60_ cage.

For BC_59_, the calculated spectrum contains a substantially larger number of active modes than pristine C_60_. Several calculated bands can be related to those observed experimentally for B-doped C_60_ films, including the calculated bands at approximately 1570, 1215, 753, 591, and 538 cm^-1^, which may be compared with experimental absorptions at 1616, 1197, 731, 576, and 545 cm^-1^, respectively. The calculated-experimental deviations for these selected modes are approximately 7-46 cm^-1^. More importantly, both spectra exhibit a greater number of bands than pristine C_60_, supporting the predicted reduction in cage symmetry and activation of additional vibrations after boron incorporation. The experimental B-doped material was produced as a thin film and included contributions from polymerized C_60_; therefore, an exact one-to-one assignment to the isolated BC_59_ model is not expected.

The calculated NC_59_ bands at approximately 1481, 1200, and 541 cm^-1^ are close to experimental C_59_HN bands at 1468, 1197, and 529 cm^-1^, with deviations of approximately 13, 3, and 12 cm^-1^, respectively. Experimental azafullerenes also show splitting and activation of the four principal C_60_-derived modes, in qualitative agreement with the more complex calculated spectrum of NC_59_. However, the experimentally observed bands at 819, 836, and 844 cm^-1^ for (C_59_N)_2_ are characteristic of coupled C-N and intercage C-C vibrations in the azafullerene dimer. These bands should therefore not be expected to correspond directly to the isolated monomeric NC_59_ model used in the present calculations.

Overall, the comparison demonstrates reasonable agreement for the principal fullerene cage vibrations and consistent symmetry-lowering trends after B or N incorporation. Nevertheless, differences in molecular composition, aggregation state, solid-state intermolecular interactions, anharmonicity, and experimental conditions account for the remaining frequency shifts and the absence of exact band-to-band correspondence. Accordingly, the experimental comparison supports the calculated vibrational trends but should not be regarded as direct experimental validation of isolated BC_59_ and NC_59_.

| Table S3. Comparison of calculated and experimental IR frequencies for pristine and heteroatom-containing fullerene systems | | | | | |
| --- | --- | --- | --- | --- | --- |
| Theoretical model | ν_Cal_ (cm^-1^) | Calculated vibrational assignment | Related experimental system | ν_Exp_ (cm^-1^) | Δν = νcal - νexp (cm^-1^) |
| C_60_ | ≈1451 | Predominantly tangential C=C stretching of the fullerene cage | Pristine C_60_ | 1428 | +23 |
| C_60_ | ≈1190 | Tangential C–C stretching of the fullerene cage | Pristine C_60_ | 1183 | +7 |
| C_60_ | ≈575 | Radial C–C cage-deformation vibration | Pristine C_60_ | 576 | -1 |
| C_60_ | ≈523 | Radial pentagonal/hexagonal cage-deformation vibration | Pristine C_60_ | 527 | -4 |
| BC_59_ | ≈1570 | Perturbed C=C stretching coupled with vibrations around the B-substitution site | B-doped C_60_ film | 1616 | -46 |
| BC_59_ | ≈1452 | Tangential C=C cage stretching perturbed by B substitution | B-doped C_60_ film | 1428 | +24 |
| BC_59_ | ≈1215 | C-C stretching coupled with local C-B-C deformation | B-doped C₆₀ film | 1197 | +18 |
| BC_59_ | ≈957 | Local C-B stretching coupled with adjacent C-C cage vibrations | B-doped C_60_ film | 1007 | −50 |
| BC_59_ | ≈753 | C-B-C bending coupled with fullerene cage deformation | B-doped C_60_ film | 731 | +22 |
| BC_59_ | ≈591 | Radial cage deformation influenced by the heavier B-containing region | B-doped C_60_ film | 576 | +15 |
| BC_59_ | ≈538 | Low-frequency C-B-C bending and cage-deformation vibration | B-doped C_60_ film | 545 | −7 |
| NC_59_ | ≈1481 | C=C cage stretching coupled with vibrations around the N-substitution site | Experimental C_59_HN | 1468 | +13 |
| NC_59_ | ≈1200 | C-C stretching coupled with local C-N-C deformation | Experimental C_59_HN | 1197 | +3 |
| NC_59_ | ≈541 | Radial cage deformation coupled with C-N-C bending | Experimental C_59_HN | 529 | +12 |
| NC_59_ | ≈541 | Radial cage deformation coupled with C-N-C bending | Experimental (C_59_N)_2_ | 525-530 | +11 to +16 |
| NC_59_ | ≈1200 | Tangential C–C stretching with contribution from the N-substitution region | Experimental (C_59_N)_2_ | 1183-1190 | +10 to +17 |
| NC_59_ | ≈1481 | Tangential C=C stretching perturbed by nitrogen substitution | Experimental (C_59_N)_2_ | 1462 | +19 |
| NC_59_ | No direct equivalent | No corresponding mode is expected for isolated monomeric NC_59_ | Experimental (C_59_N)_2_ | 819, 836 and 844 | — |

The alternative optimized configurations shown in Figure S3 exhibit less negative adsorption energies than the configurations selected for the subsequent analyses in the manuscript. Their Eads values of -6.65, -7.09, and -13.65 kcal.mol^-1^ are substantially less favorable than the corresponding values reported for the selected C_60_@Furan, NC_59_@Furan, and BC_59_@Furan structures. Therefore, provided that all energies were calculated at the same theoretical level, these geometries represent less stable local minima and were not selected for the detailed electronic-structure and bonding analyses. Figure S4 also shows a qualitative comparison of the reported dipole moment values.

Fig S3. Initial and fully optimized geometries of furan adsorption on C_60_, NC_59_, and BC_59_, shown from left to right. The arrows indicate structural relaxation from the selected initial C-H-directed configurations to the corresponding optimized complexes.

Fig S4. Comparison of the calculated dipole moment with computational functionals (A) B97D and (B) WB97XD.
